# Overcoming the Limitations of Organocatalyzed Glycolysis of Poly(ethylene terephthalate) to Facilitate the Recycling of Complex Waste Under Mild Conditions

**DOI:** 10.1021/acsapm.4c00326

**Published:** 2024-03-22

**Authors:** Ion Olazabal, Emelin J. Luna Barrios, Steven De Meester, Coralie Jehanno, Haritz Sardon

**Affiliations:** † POLYMAT, University of the Basque Country UPV/EHU, Joxe Mari Korta Center, Avda. Tolosa 72, 20018 Donostia-San Sebastián, Spain; ‡ Department of Green Chemistry and Technology, Ghent University, Graaf Karel De Goedelaan 5, Kortrijk 8500, Belgium; § POLYKEY, Avda. Tolosa 72, 20018 Donostia-San Sebastian, Spain

**Keywords:** organocatalysis, chemical recycling, complex waste, glycolysis, poly(ethylene terephthalate)

## Abstract

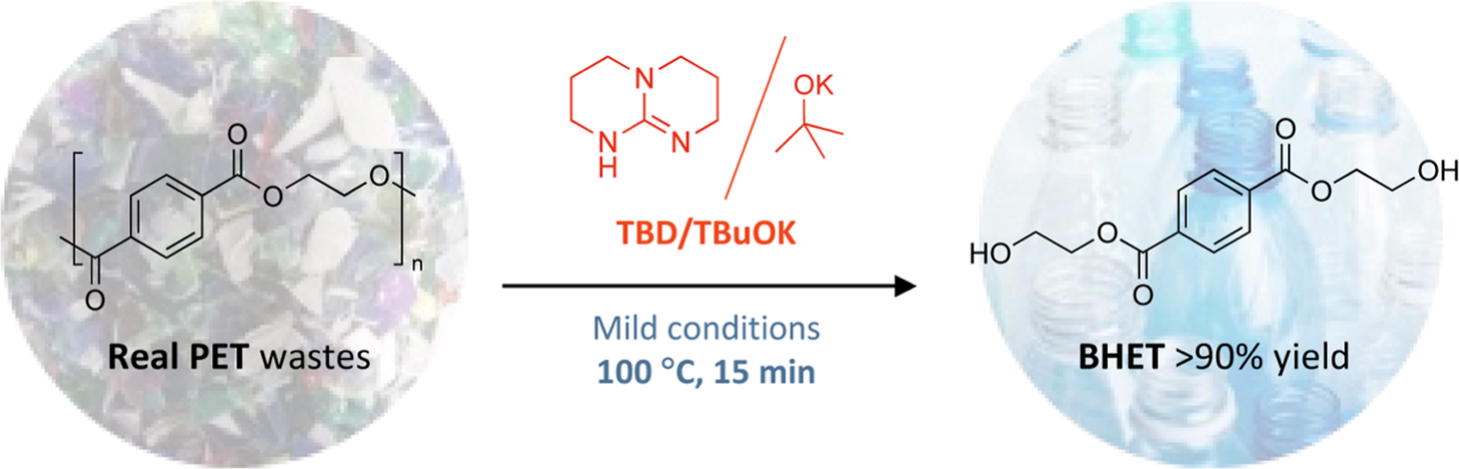

Although multiple methods have been reported in the literature for the chemical recycling of poly(ethylene terephthalate) (PET), large-scale depolymerization is not yet widely employed. The main reasons for the limited adoption of chemical recycling of PET are the harsh conditions required and the lack of selectivity. In this study, the organocatalytic glycolysis of PET mediated by organic bases at low temperatures is studied, and routes to avoid the deactivation of the catalyst are explored. It is shown that the formation of terephthalic acid by uncontrolled hydrolysis leads to issues which can be resolved using potassium *tert*-butoxide as a cocatalyst. Finally, complex PET waste obtained from a mechanical recycling plant was depolymerized under optimized conditions, obtaining bis(2-hydroxyethyl) terephthalate yields >90% in less than 15 min at only 100 °C. These results open the way to efficient recycling of PET-enriched waste streams under milder conditions.

## Introduction

Poly(ethylene terephthalate) (PET) is one of the most widely produced plastics worldwide due to its excellent mechanical, thermal and barrier properties, in addition to its low cost.^1^ Because it is a thermoplastic material, mechanical recycling is the most widely used method for PET.^2^ Even though it is a well-implemented cost-efficient technology, when contaminants are present in the waste stream (metals, additives, organic wastes, polyolefins, ...) structural degradation of the PET can occur, which forces the mechanical recycler to choose between the treatment of a maximum quantity of waste and a high quality recycled PET (rPET).^3^ Using current methods, once involved in the process of mechanical recycling, the sorting, cleaning, and gridding procedure applied to PET waste leads to a loss of up to 55% of the PET entering a recycling plant depending on the origin of the feedstock.^4^ PET mixed with other plastics, colored PET, PET with labels, PET mixed with metal, PET-based textiles, and much more are removed from the recycling line during the process to maintain the final quality of rPET. As an alternative, selective chemical recycling, in which PET is depolymerized into either monomers or added-value chemicals, is expected to be more suitable for complex waste streams that are currently unfit for mechanical recycling.^5−8^

Multiple approaches have been already reported for the depolymerization of PET including hydrolysis,^9,10^ methanolysis,^11−13^ or glycolysis,^14−16^ giving rise to different depolymerization products such as terephthalic acid (TPA), dimethyl terephthalate, or bis(2-hydroxyethyl) terephthalate (BHET). Among these methods, glycolysis is currently the most studied, as BHET is a common monomer for PET production, and the reaction only requires a catalyst and ethylene glycol, which is one of the monomers of PET.^17−20^ To date, numerous reports describe the glycolysis of PET under various conditions with different catalytic systems.^21−24^ Among the available catalysts utilized in the open literature, nitrogen-based organocatalysts, such as 1,5,7-triazabicyclo[4.4.0]dec-5-ene (TBD), 1,8-diazabicyclo 5.4.0 undec-7-ene (DBU), or 4-dimethylaminopyridine (DMAP), have been widely studied.^25,26^ With TBD as the catalyst, full degradation of the polymer can be obtained in minutes using ethylene glycol in excess at 190 °C under a nitrogen atmosphere. More recent reports, including publications from our group, have explored the use of thermally resistant acid/base complexes^27−30^ while other organocatalytic routes, mainly using metal-free ionic liquids, have also been explored.^31−35^ However, these methodologies usually require high temperatures, over 180 °C, to reach complete depolymerization on an acceptable time scale.

Lowering the temperature of the reaction of an alcoholysis is usually only permitted through the use of highly energetic conditions, which includes microwave irradiation for example.^36−38^ However, very recently, it has been demonstrated that the combination of a good solvent for PET and a specific catalytic system allows the temperature of the process to be reduced to 120 °C. For example, Tanaka et al. have reported the depolymerization of PET catalyzed by alkali metal methoxide (particularly lithium salts) through methanolysis under mild temperatures.^39^ Dimethyl carbonate was used as a trapping agent to obtain depolymerization in 5–6 h, leading to ethylene carbonate as a byproduct. Pham et al. described a catalytic route for the methanolysis of PET using potassium carbonate as catalyst, and the effects of cosolvents on the catalytic performance were investigated.^11^ While complete depolymerization was achieved, it required long reaction times (typically >24 h). Most recently, our group has also demonstrated that the selection of an appropriate solvent can allow operating temperatures as low as 65 °C which reduces the CO_2_ footprint by at least 20% compared to the same reaction in bulk.^40^ Although the specific role of the solvent in lowering the operating temperature is still not clear, we hypothesized that it could facilitate the diffusion of the nucleophile into the polymer chains, which lowers the energetic barrier of the depolymerization process.

In this work, organic bases have been explored as catalyst in the solvent-assisted glycolysis of PET at low temperatures. Based on results obtained in a previous study of our group for the organocatalytic depolymerization of bisphenol A-polycarbonate (BPA-PC) under similar conditions, 1-methylimidazole was selected as solvent considering the structural similarities between PET and BPA-PC.^41^ First, different bases are explored as catalyst for the potential low-temperature glycolysis of PET. In an attempt to lower the amount of catalyst that is required for the depolymerization, the use of a cocatalyst system is explored to mitigate the effect of residual water on the depolymerization. ^1^H NMR spectroscopy analysis shows that residual water present in the reaction medium promotes the formation of TPA, which deactivates the TBD catalyst. To overcome this issue, a sterically hindered, high p*K*_A_ base, i.e., potassium *tert*-butoxide (*t*BuOK), was added to neutralize the formed TPA. It is shown that the addition of *t*BuOK maximizes the monomer yield obtained from real PET waste, thus overcoming the negative impact of water in the depolymerization process.

## Results and Discussion

**Figure 1:**
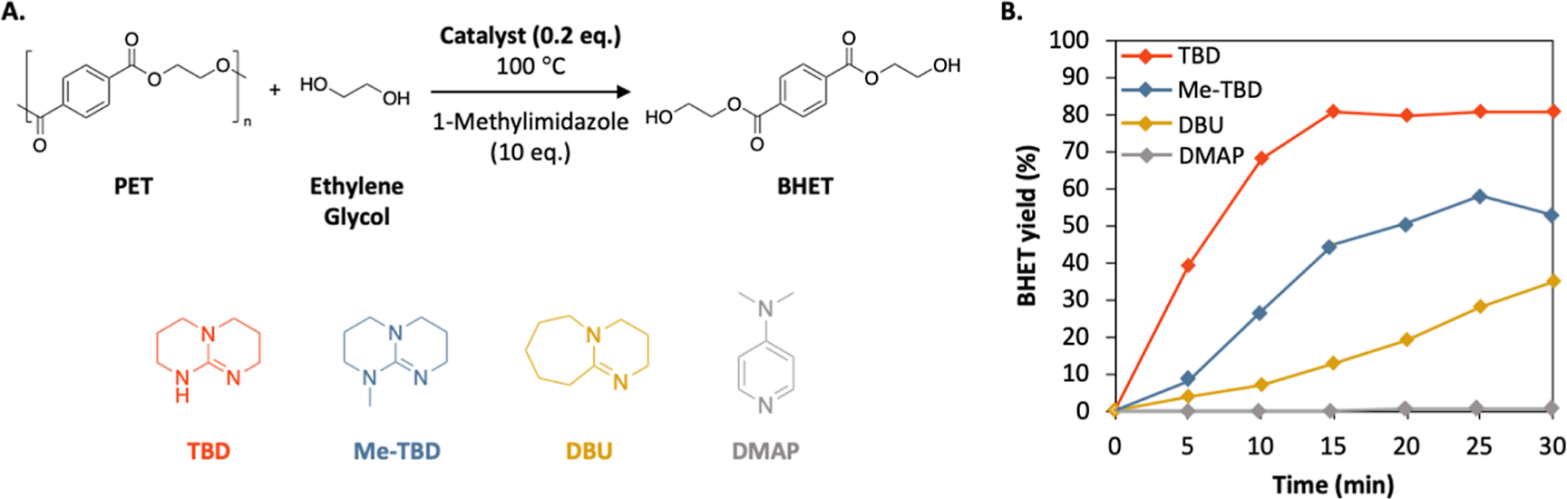
(A) Scheme for the depolymerization of PET with ethylene glycol as a nucleophile (3 equiv) in 1-methylimidazole (10 equiv) with 0.2 equiv of catalyst at 100 °C and (B) kinetics of the reaction with different organic bases as catalyst (0.2 equiv) (Figures S3–S6).

To perform the organocatalytic depolymerization of PET at low temperatures on a reasonable time scale, an adequate solvent and a strong catalyst are required. Based on the results obtained with the Hansen solubility parameters theory in previous studies of our group, 1-methylimidazole was selected as solvent^41^ while the most common organic bases (i.e., DMAP, TBD, 7-methyl-1,5,7-triazabicyclo[4.4.0]dec-5-ene (Me-TBD), and DBU) were screened for the reaction (Figure 1A). The reaction was performed with ethylene glycol in excess (3 equiv) in 10 equiv of 1-methylimidazole with 0.2 equiv of catalyst at 100 °C for 30 min. Screenings of the ethylene glycol and 1-methylimidazole loads were performed to determine the optimized quantities of both reagent and solvent (Figures S1 and S2). The reaction kinetics was monitored by ^1^H NMR spectroscopy in DMSO-*d*_6_.

The results obtained show that the choice of base plays an important role in the depolymerization. DMAP, which is the organic base with the lowest p*K*_A_ (17.95 in acetonitrile^26^), was unable to catalyze the depolymerization of PET in 30 min. This result could be expected considering that even at higher temperatures (>180 °C) with the use of DMAP, no depolymerization occurred. On the contrary, DBU, Me-TBD, and TBD, all of which have higher basicity—p*K*_A_ = 24.31, 26.02, and 25.47, in acetonitrile, respectively^26,42,43^—promote the depolymerization of PET. By weighing the residual PET recovered after filtration, conversion rates of 91% with TBD, 57% with Me-TBD, and 38% with DBU were obtained. BHET yields calculated through the integration of the characteristic signals of the molecule in the ^1^H NMR spectra are close to the depolymerization rate for all catalysts, i.e., 81, 53, and 36%, respectively. The difference between the conversion rate of PET and the BHET yield is explained by the identification of 10 and 4% of dimers, for TBD and Me-TBD, respectively, in the NMR spectra (Figures S3 and S4, characteristic signal at δ = 4.28 ppm). These results suggest that the basicity of the catalyst is not the only determining factor for obtaining the best depolymerization performance (Figure 1B). It may be noted that a previous report on the glycolysis of PET at higher temperatures—i.e., 180 °C—in bulk did not notice such significant differences between organic bases with similar p*K*_A_.^26^ The obtained data led us to investigate the reasons for these significant differences between the catalysts.

**Scheme 1:**
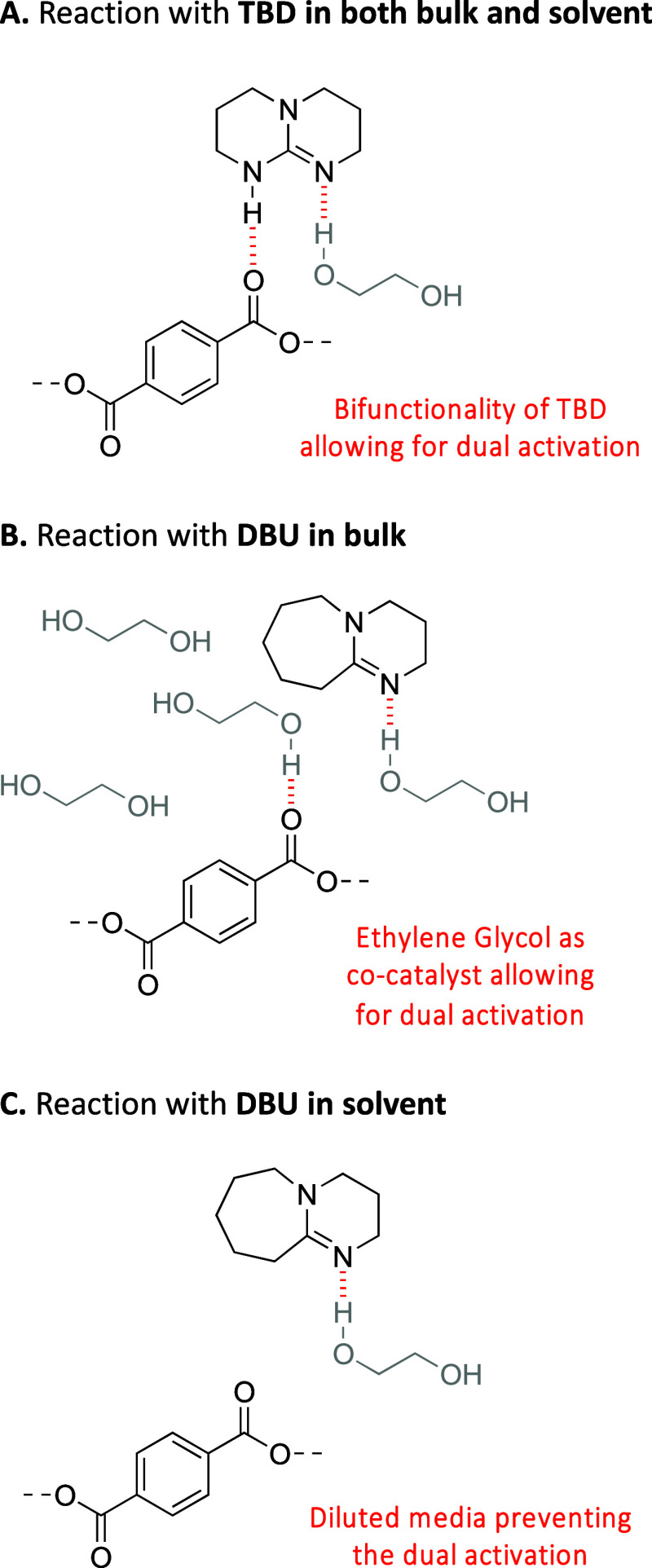
Mechanistic Difference between the Glycolysis of PET (A) With TBD as catalyst in both bulk and diluted conditions, (B) with DBU in bulk, and (C) in solution.

It has been proven that the depolymerization of PET mediated by organic bases occurs through a dual H-bonding mechanism in which both the oxygen of the carbonyl group of the ester and the hydrogen of the alcohol are activated.^25^ Although DBU does not present any labile proton that allows for the dual activation to occur, Horn et al. explained the similar behavior of TBD and DBU at high temperatures through computational investigations by demonstrating that ethylene glycol, used as nucleophile and solvent, contributes to the depolymerization mechanism as a cocatalyst.^44^ For TBD, the dual activation mechanism is ensured by the imine, which activates the nucleophile, and the proton of the secondary amine, which activates the carbonyl group of PET (Scheme 1A). With DBU, the imine also activates the nucleophile, while the hydroxyl of a molecule of ethylene glycol activates the carbonyl (Scheme 1B). We hypothesized that in the context of solvent-assisted depolymerization, the dilute conditions do not allow for the ethylene glycol to assist the catalytic process. This does not affect the catalytic performance of TBD, as its bifunctional nature allows for catalysis of the reaction through a dual H-bonding mechanism even in the absence of ethylene glycol. However, with DBU as a catalyst, if the ethylene glycol does not participate in the catalytic process because of the dilute conditions, efficient dual activation cannot occur and lower depolymerization rates are obtained (Scheme 1C). The same argument can be applied to Me-TBD, in which the lack of a secondary amine in the structure leads to inferior catalytic performances, with a BHET yield at 30 min similar to what was obtained for DBU, 45 vs 38%.

**Figure 2:**
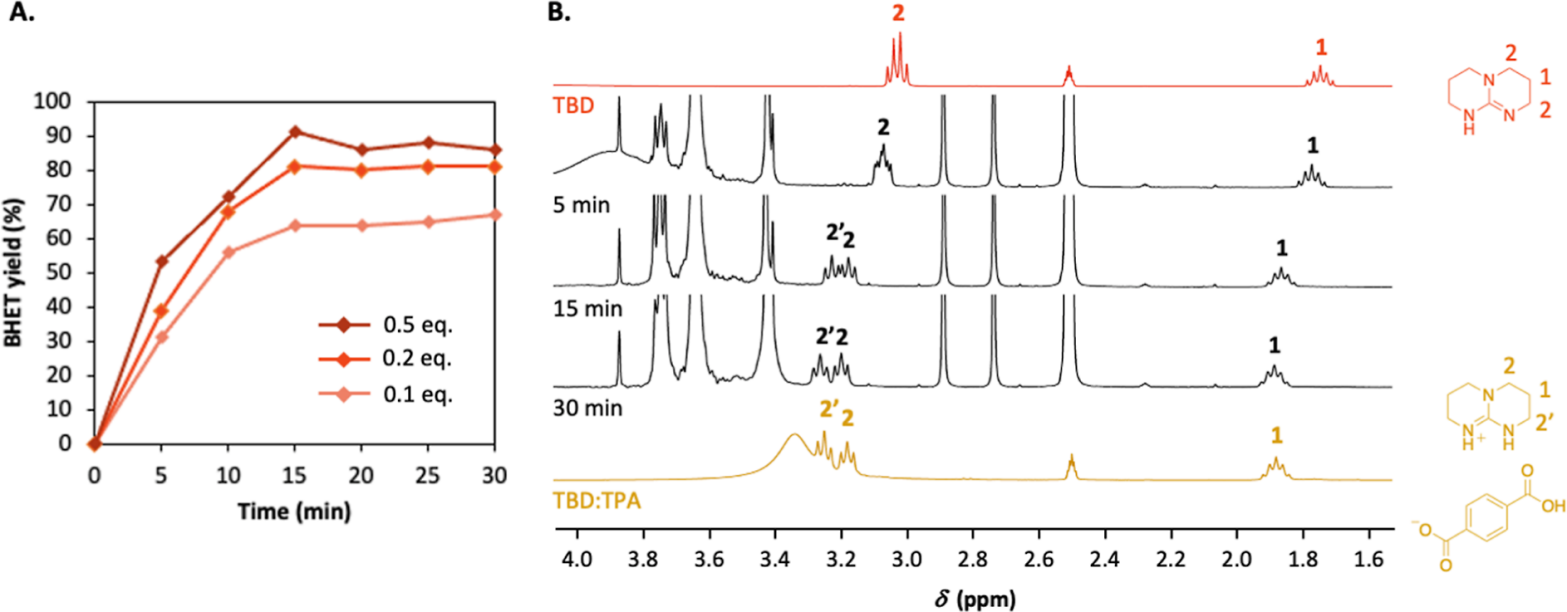
(A) Kinetics of the depolymerization of PET with ethylene glycol (3 equiv) in 1-methylimidazole (10 equiv) at 100 °C with different catalyst loading and (B) stacked ^1^H NMR spectra of lone TBD, aliquot of the depolymerization reaction with 0.2 equiv of TBD after 5, 15, and 30 min, and salt formed out of an equimolar mixture of TBD and TPA (Figures S8–S10).

Given that TBD displayed the best results at 100 °C, different catalyst loads were investigated with the aim of further increasing the final yield of BHET (Figure 2A). Employing 0.1 equiv of TBD, only 60% of BHET was obtained, while 0.2 equiv allowed for an increase of the yield up to 81%. Increasing the catalyst load further to 0.5 equiv only led to a slight further increase, reaching 88%. In all three reactions, a plateau was reached after 15 min of reaction, indicating that the depolymerization reached an equilibrium, which suggests the deactivation of the catalyst. The ^1^H NMR spectra revealed a gradual change in the characteristic signals of TBD over time. The characteristic signals for the four –CH_2_ groups in the α position (Figure 2B, **2**) to the nitrogen atoms changed from a single quadruplet at δ = 3.03 ppm to two well-defined triplets at δ = 3.26 and 3.20 ppm. Concomitantly, the quintuplet signal, which is characteristic of the –CH_2_ in the β position to the nitrogen atoms, shifted from δ = 1.75 to 1.88 ppm (Figure 2B, **1**). This shift to higher positions and the splitting into two triplets for the hydrogen closest to the nitrogen atoms is typical of the formation of a salt between TBD and an acid.^27^ When TBD is in a complex with a weak acid, the catalytic activity can decrease, preventing further reaction, especially at temperatures as low as 100 °C, where the hydrogen bonding mechanisms are important.

In the present reaction, it is suspected that the possible salt could be formed from TBD and TPA resulting in the undesired hydrolysis of PET due to residual water. The comparison of the ^1^H NMR spectrum of the reaction after 30 min with the spectrum of the model salt TBD:TPA corroborates this hypothesis, as the signals are identical, in terms of both multiplicity and shifting. To corroborate the strong affinity between the catalyst and the polymer, a mixture of 1 equiv of PET with 1 equiv of TBD in 10 equiv of methylimidazole was analyzed through a DOSY NMR experiment. Compared with the value in the NMR spectra of only TBD, i.e., from 3.13 × 10^–5^ cm^2^/seg, the diffusion coefficient has changed for 6.91 × 10^–6^ cm^2^/seg which is the same value as PET (Figure S7). These similar values in diffusion highlight the strong interaction between TBD and the terephthalate moieties.

**Figure 3:**
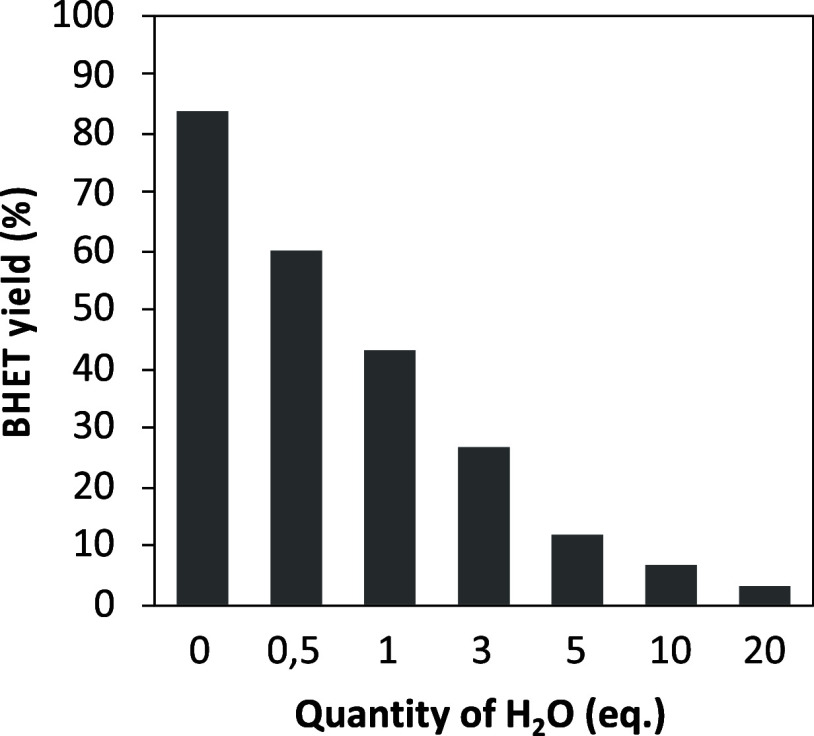
Impact of the water content on the BHET yield in the TBD-catalyzed glycolysis of PET (Figures S11–S17).

**Figure 4:**
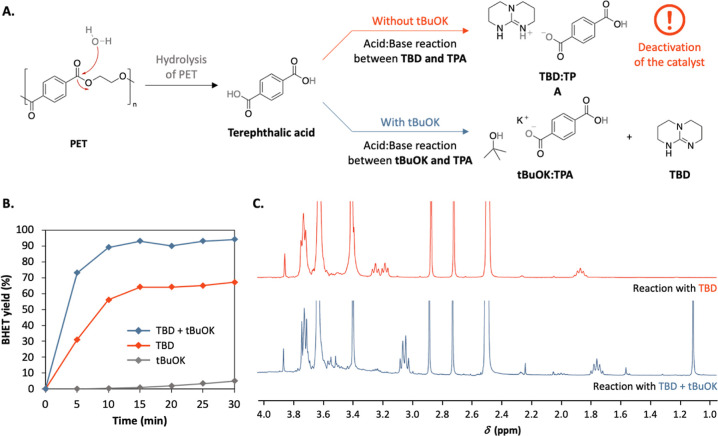
(A) TBD catalyzed depolymerization process with and without *t*BuOK showing the deactivation of the TBD catalyst through an acid–base reaction with TPA arising from the hydrolysis of PET. (B) Kinetics of the depolymerization of PET with ethylene glycol (3 equiv) in 1-methylimidazole (10 equiv) at 100 °C catalyzed by 0.2 equiv of *t*BuOK (gray), 0.1 equiv of TBD (red) and 0.1 equiv of TBD + 0.2 equiv of *t*BuOK (blue) and (C) ^1^H NMR spectra of the crude product for the reactions with TBD and the system TBD + *t*BuOK (Figures S3, S18, and S19).

Our hypotheses were that the residual PET is contaminated with traces of water, thus leading to the formation of TPA as a byproduct. To investigate this hypothesis, we evaluated the impact of water on the reaction, and the results were analyzed by ^1^H NMR spectroscopy. As expected, the BHET yield gradually decreased as the water content increased (Figure 3). In the absence of water, 84% yield of BHET was obtained when 1 equiv of water was added, the BHET yield dramatically decreased to 42%. Signals characteristic of both carboxylate-terminated PET oligomers and TPA were observed in the aromatic region (δ = 8.02–7.90 ppm). Higher water content continued to hinder the depolymerization reaction, with a 12% yield of BHET when 5 equiv of water was added to the reaction and less than 7% yield of BHET obtained with 10 equiv of water. These results confirmed that traces of water can initiate the depolymerization of PET into TPA which later reacts with TBD to form the TBD:TPA complex, which significantly reduces the catalytic performance of TBD and consequently lowers the BHET yield (Figure 4A). From an industrial perspective, the necessity of using extremely dry conditions, i.e., to eliminate all traces of water from the PET waste, ambient air, and all other reagents employed, would significantly complicate the process and inhibit scale-up. Therefore, a simpler strategy was applied to avoid the deactivation of the catalyst. The central idea is that instead of trying to inhibit the hydrolysis of PET, the formation of a salt between TPA and the catalyst would be avoided by adding a stronger base, which would form a more stable complex with the residual TPA (Figure 4A). *t*BuOK (p*K*_A_ in water = 17), which has the highest p*K*_A_ of the alkoxides, was thus used as a cocatalyst with TBD (p*K*_A_ in water = 15.2^42^). Besides being a strong base, the steric hindrance of *t*BuOK makes it a non-nucleophilic base, which should prevent undesired transesterification reactions and the formation of side products during the depolymerization process. The depolymerization of PET was performed under the following conditions: 0.1 equiv of TBD, 1-methylimidazole as a solvent, 100 °C, but with an additional 0.2 equiv of *t*BuOK and was monitored through ^1^H NMR spectroscopy (Figure 4B). After only 15 min of reaction, the depolymerization of PET was completed and the BHET yield increased to 95% while the reactions with TBD (0.1 equiv) and *t*BuOK (0.2 equiv) individually only provided 67% and 5% yield of BHET, respectively. As expected, in the ^1^H NMR spectra, the dissociation of the TBD signals corresponding to the formation of a salt with TPA (δ = 3.28–3.15 ppm) was not observed when the *t*BuOK was added to the reaction (Figure 4C).

**Figure 5:**
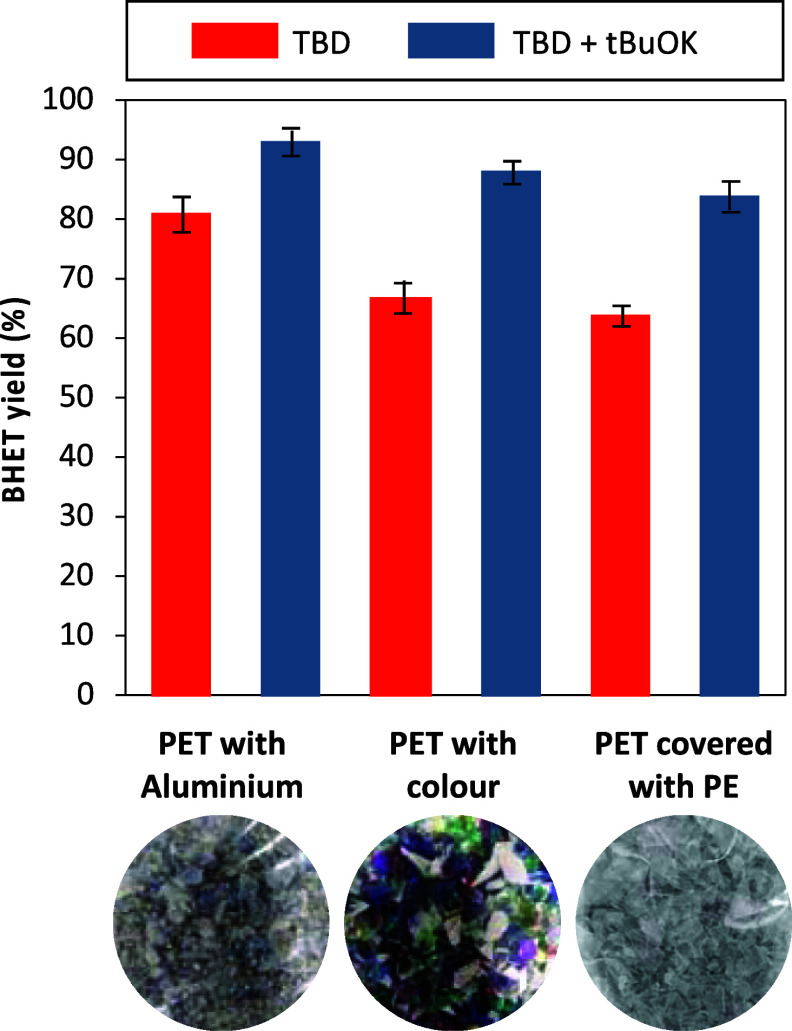
Comparison of BHET yield from the depolymerization of PET with TBD or with TBD and potassium *tert*-butoxide as a catalyst for different real PET waste. Reaction conditions: PET waste (1 equiv), 1-methylimidazole (10 equiv), ethylene glycol (3 equiv), TBD (0.1 equiv), or TBD + *t*BuOK (0.1 + 0.2 equiv), 100 °C (Figures S20 to S25).

To demonstrate the effectiveness of the TBD/*t*BuOK catalytic mixture, we evaluated the depolymerization reaction in different types of nonmechanically rPET wastes. The PET mixtures employed for testing the present system on “real waste” were provided by a mechanical recycling plant. The different samples are considered too complex to obtain rPET of sufficient quality and are currently discarded during the mechanical recycling process. This includes PET mixed with metals (mainly aluminum), colored PET (a mixture of organic and inorganic dyes), and multilayer materials (constituted of a main layer of PET covered by a thin layer of polyethylene). The TBD/*t*BuOK system was compared to the TBD-catalyzed depolymerization without *t*BuOK. The reaction was performed under the optimized conditions from the previous section, i.e., ethylene glycol (3 equiv), TBD (0.1 equiv), and *t*BuOK (0.2 equiv), 100 °C in 1-methylimidazole (10 equiv) (Figure 5).

The BHET yield was determined through ^1^H NMR spectroscopy with DMF as internal standard. The results obtained show that using only TBD, the yield of BHET ranges from 63% for PET-containing multilayer materials to 81% for PET mixed with aluminum. When *t*BuOK is added to the reaction, substantial improvements were noticed, with BHET yields reaching 85, 87, and 92% for PET-containing multilayer materials, colored PET, and PET mixed with aluminum, respectively. These experiments demonstrate the tolerance of the method toward potential contamination present in real samples. The addition of *t*BuOK enhances the catalytic performance of TBD, as it limits the formation of a TBD complex with TPA and allows the depolymerization of contaminated samples under mild conditions.

## Conclusions

This study demonstrates that the glycolysis of PET can be performed under mild conditions. Through an initial study using different common organic bases, it was found that TBD is an efficient catalyst for the solvent-assisted depolymerization of PET at a lower temperature, i.e., 100 °C, with BHET yields >80% after only 15 min in 1-methylimidazole. However, the deactivation of TBD during the reaction prevents the depolymerization from reaching higher BHET yields as a result of trace amounts of water, which leads to the formation of a salt between the catalyst and residual TPA obtained from hydrolysis. To solve this issue, potassium *t*BuOK, a high p*K*_A_ and non-nucleophilic base, was added to the system to neutralize the TPA and maintain the catalytic performance of TBD. In the presence of potassium *t*BuOK higher conversions and higher BHET yields were obtained, up to 92%, including in real PET waste samples. Further studies are now being performed to implement this process in multimaterial PET-based waste streams.

## Supporting Information Available

^1^H NMR spectrum and DOSY experiments of the different depolymerization reactions (PDF)
