# Supplementary material for: Overcoming the Limitations of Organocatalyzed Glycolysis of Poly(ethylene terephthalate) to Facilitate the Recycling of Complex Waste Under Mild Conditions
Source: ACS Appl Polym Mater. 2024 Mar 22;6(7):4226–32. doi: 10.1021/acsapm.4c00326 (PMC11019730; doi:10.1021/acsapm.4c00326)
Supplement: Supplementary file 1 — ap4c00326_si_001.pdf [file ap4c00326_si_001.pdf]

# Supporting Information

## Overcoming the Limitations of Organocatalysed Glycolysis of poly(ethylene terephthalate) to Facilitate the Recycling of Complex Wastes under Mild Conditions

Ion Olazabal[1], Emelin J. Luna Barrios[1,2], Steven De Meester[2], Coralie Jehanno[1,3*], Haritz Sardon[1*]

*[1]POLYMAT, University of the Basque Country UPV/EHU, Avda. Tolosa 72, 20018 Donostia-San Sebastian (Spain)*
*[2]Department of Green Chemistry and Technology, Ghent University, Graaf Karel De Goedelaan 5, Kortrijk, 8500 (Belgium)*
*[3]POLYKEY, Avda. Tolosa 72, 20018 Donostia-San Sebastian (Spain)*

# Experimental section

## Materials

Post-consumer bottle grade PET were used without any purification. In case of the materials obtained directly from water bottles, they were cut in small pieces and dried to eliminate water traces. Triazabicyclo[4.4.0]dec-5-ene (TBD), 7-Methyl-1,5,7-triazabicyclo[4.4.0]dec-5-ene (Me-TBD), 1,8-Diazabicyclo[5.4.0]undec-7-ene (DBU), 4-(Dimethylamino)pyridine (DMAP), ethylene glycol, 1-methylimidazole, terephthalic acid, potassium *tert*-butoxide and anhydrous dimethyl formamide (DMF) were purchased from Sigma Aldrich and were used without further treatment.

**The samples of PET wastes containing metal, PE and colourants were kindly provided by the company Recopolymers (https://www.recopolymers.com/) specialised in mechanical recycling.**

## General procedure for the catalytic degradation of PET

In each experiment, 0.5 g of PET flakes were degraded using ethylene glycol with a certain amount of catalyst in the presence of methylimidazole as solvent and 0.5 eq. of dry DMF as internal pattern. For all the reactions, reagents, solvent and catalyst were loaded in a 10 mL vial equipped with a magnetic stirrer which was introduced in an oil bath pre-heated at the desired temperature. The depolymerisations were carried out under atmospheric pressure at 100 °C for a time period of 30 min. At completion of the reaction, the crude product was cooled down to 50 °C to separate through filtration the eventual unreacted PET and solid contaminants before colling down the liquid phase to room temperature and adding of a large excess of water. The resulting solution was vigorously stirred and filtered to separate ethylene glycol, catalyst and main product from oligomers, insoluble in water. The aqueous transparent filtrate was stored in a refrigerator at 4 °C overnight. White needle-like crystals were formed in the solution, which were collected and dried over vacuum prior to characterisation by means of $^{1}H$ NMR spectroscopy (400 MHz, DMSO-$d_6$) $\delta$ (ppm) 8.12, (s, 4H, CH), 4.97 (t, 2H, OH), 4.32 (t, 4H, O–$CH_2$), 3.73 (q, 4H, $CH_2$–OH).

The **depolymerisation rate** was determined at the end of the reaction by weighting the unreacted PET after separation from the crude and drying for 24 h in a vacuum oven at 60 °C.

Unless another methodology is mentioned, **BHET yield** was calculated considering the integration of the characteristics signals of the aromatic group ($\delta$ = 8.12 ppm (s, 4H, CH)), comparing with the signal of DMF ($\delta$ = 2.73 ppm (s, 3H, $CH_3$)), added as internal standard. All spectra were recorded employing DMSO-$d_6$ provided by Eurisotop.

## Specific procedure for calculating the depolymerisation rate and BHET yield of the PET wastes

For ensuring repeatability, all experiments conducted with complex PET wastes were performed **three times**.

**With metal →** For ensuring repeatability in the experiments, the samples containing aluminium were sorted by hand and reassembled for strictly obtaining 50% of metal and 50% of PET, by weight. Conversion and BHET yield were calculated according to the abovementioned method.

**With PE layer →** The PE representing around 5% of the weight of the sample (data provided by the company which has provided the samples Conversion and BHET yield were calculated according to the abovementioned method.

**With colour** → No specific precautions should be taken for the PET with colours, the quantity of colourants and additives being neglectable (in weight), compared to the quantity of PET.

## Characterisation methods

$^1H$ NMR spectra were recorded at room temperature with Bruker Avance DPX 300 or Bruker Avance 400 spectrometers using deuterated DMSO as solvent. The chemical shifts were reported as $\delta$ in parts per million (ppm) relative to the traces of non-deuterated solvent (*e.g.* $\delta$ = 2.50 ppm for DMSO-$d_6$). Data were reported as: chemical shift, multiplicity (s = singlet, d = doublet, t = triplet, m = multiplet, br = broad), coupling constants (J) given in Hertz (Hz), and integration.

# Screening of parameters for the depolymerisation reactions

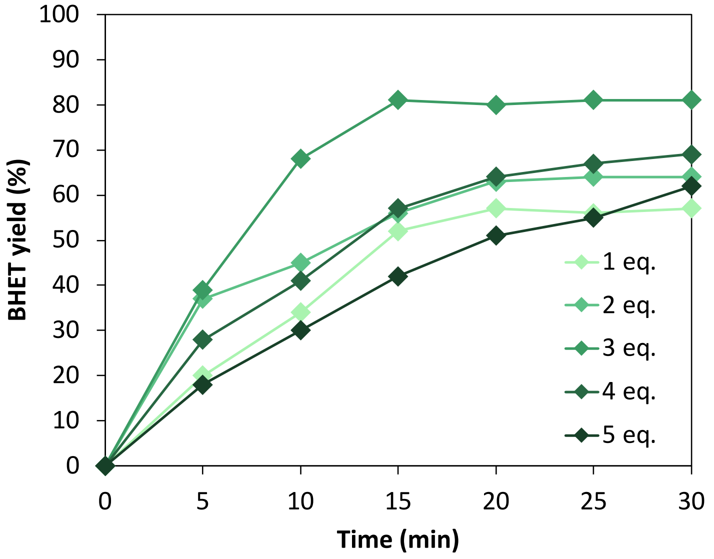


**Figure S1.** Kinetics of the reaction with different ethylene glycol loads with 0.2 eq. of TBD as catalyst and 10 eq. of 1-methylimidazole as solvent, at 100 °C. (400 MHz, DMSO-$d_6$, 298 K)

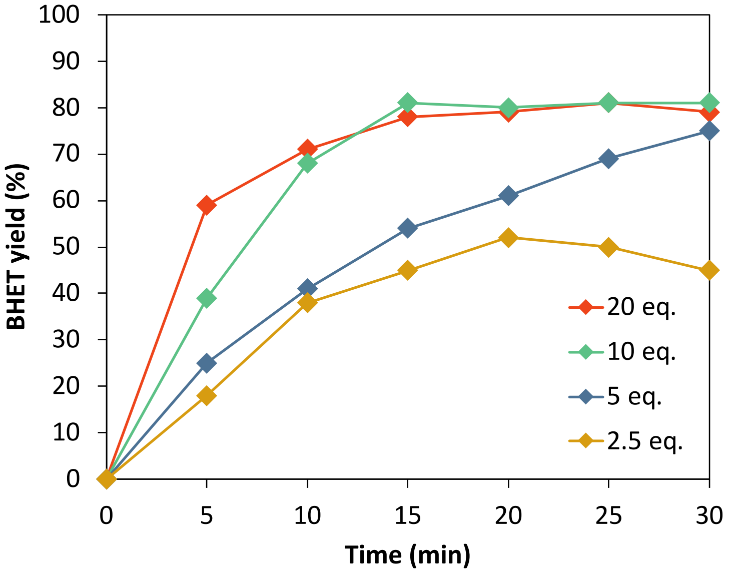


**Figure S2.** Kinetics of the reaction with different 1-methylimidazole loads with 3 eq. of ethylene glycol and 0.2 eq. of TBD as catalyst at 100 °C. (400 MHz, DMSO-$d_6$, 298 K)

# Kinetics of the depolymerisation reactions with various catalysts

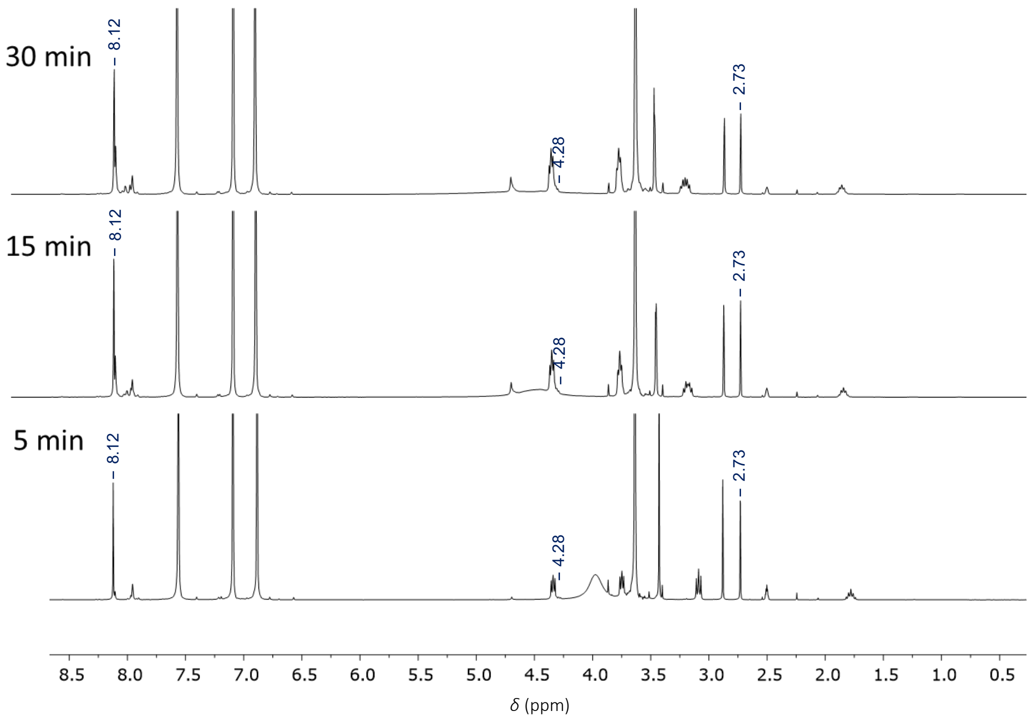


**Figure S3.** Kinetics of PET glycolysis with 3 eq. of ethylene glycol as reagent, **0.2 eq. of TBD** as catalyst and 10 eq. of 1-methylimidazole as solvent, at 100 °C. (400 MHz, DMSO-$d_6$, 298 K) The BHET and dimer yields were evaluated by comparing the integration of their characteristic signals ($\delta$ = 8.12 ppm (s, 4H, CH)) and ($\delta$ = 4.27 ppm (t, 4H, O-$CH_2$)), respectively, with the characteristic signals of DMF as internal standard ($\delta$ = 2.73 ppm (s, 3H, $CH_3$)).

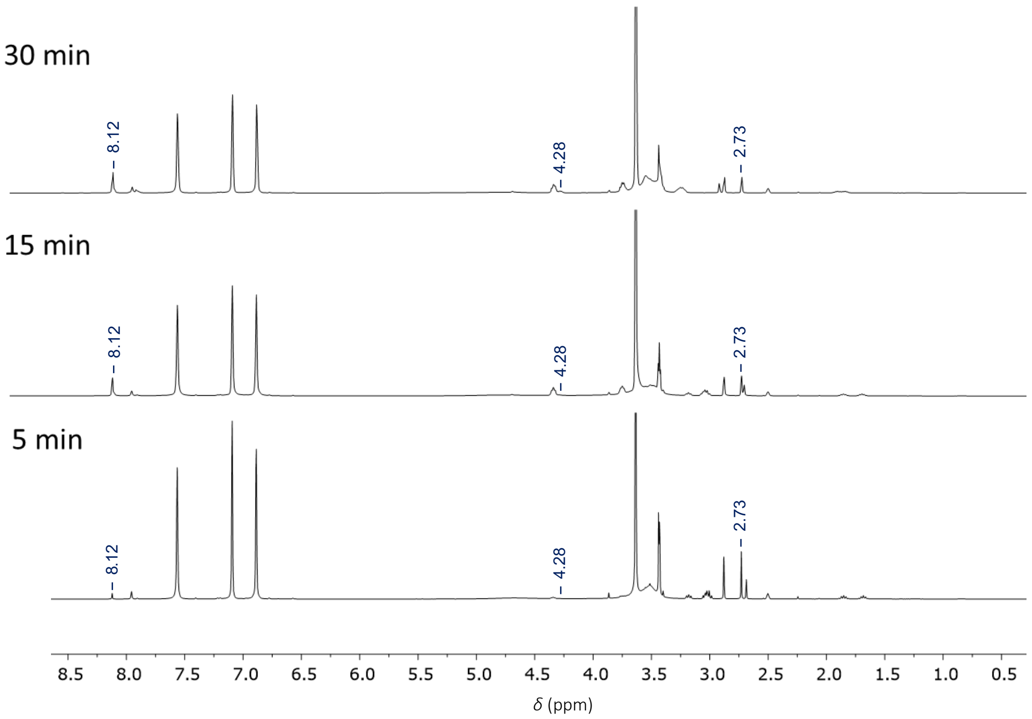


**Figure S4.** Kinetics of PET glycolysis with 3 eq. of ethylene glycol as reagent, **0.2 eq. of Me-TBD** as catalyst and 10 eq. of 1-methylimidazole as solvent, at 100 °C. (400 MHz, DMSO-$d_6$, 298 K) The BHET and dimer yields were evaluated by comparing the integration of their characteristic signals ($\delta$ = 8.12 ppm (s, 4H, CH)) and ($\delta$ = 4.27 ppm (t, 4H, O-$CH_2$)), respectively, with the characteristic signals of DMF as internal standard ($\delta$ = 2.73 ppm (s, 3H, $CH_3$)).

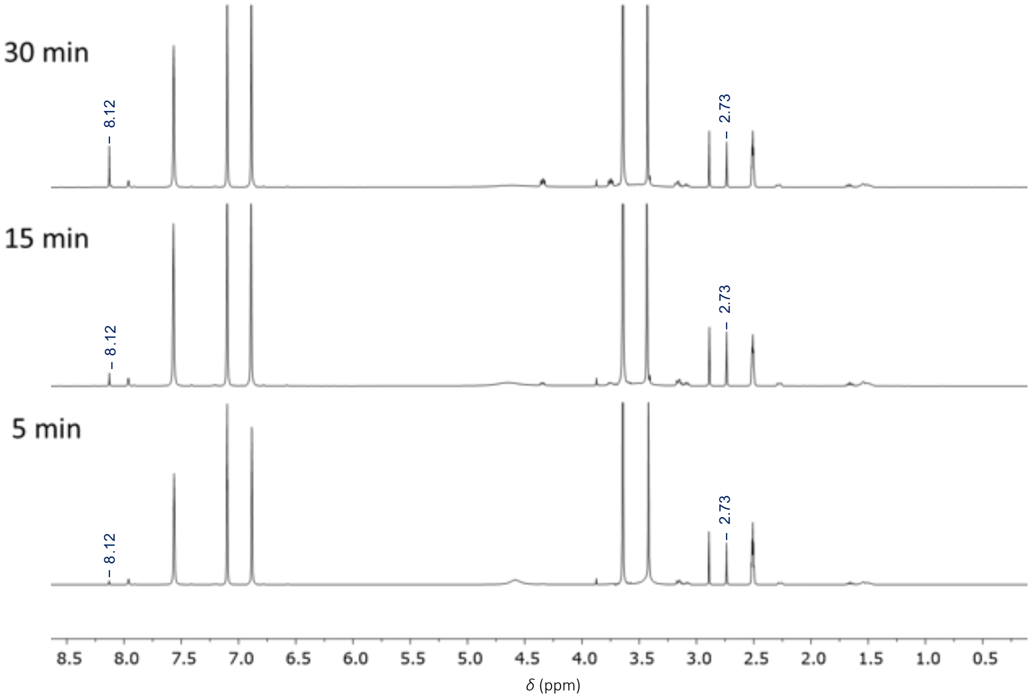


**Figure S5.** Kinetics of PET glycolysis with 3 eq. of ethylene glycol as reagent, **0.2 eq. of DBU** as catalyst and 10 eq. of 1-methylimidazole as solvent, at 100 °C. (400 MHz, DMSO-$d_6$, 298 K) The BHET yield was evaluated by comparing the integration of its characteristic signals ($\delta$ = 8.12 ppm (s, 4H, CH)) with the characteristic signals of DMF as internal standard ($\delta$ = 2.73 ppm (s, 3H, $CH_3$)).

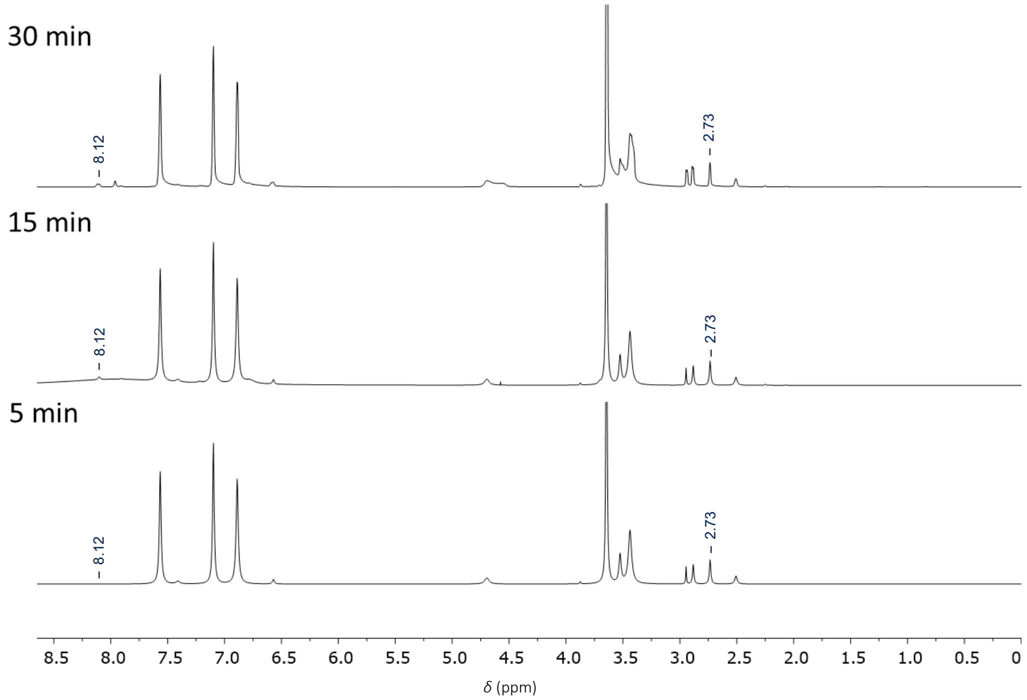


**Figure S6.** Kinetics of PET glycolysis with 3 eq. of ethylene glycol as reagent, **0.2 eq. of DMAP** as catalyst and 10 eq. of 1-methylimidazole as solvent, at 100 °C. (400 MHz, DMSO-$d_6$, 298 K) The BHET yield was evaluated by comparing the integration of its characteristic signals ($\delta$ = 8.12 ppm (s, 4H, CH)) with the characteristic signals of DMF as internal standard ($\delta$ = 2.73 ppm (s, 3H, $CH_3$)).

# DOSY NMR experiments

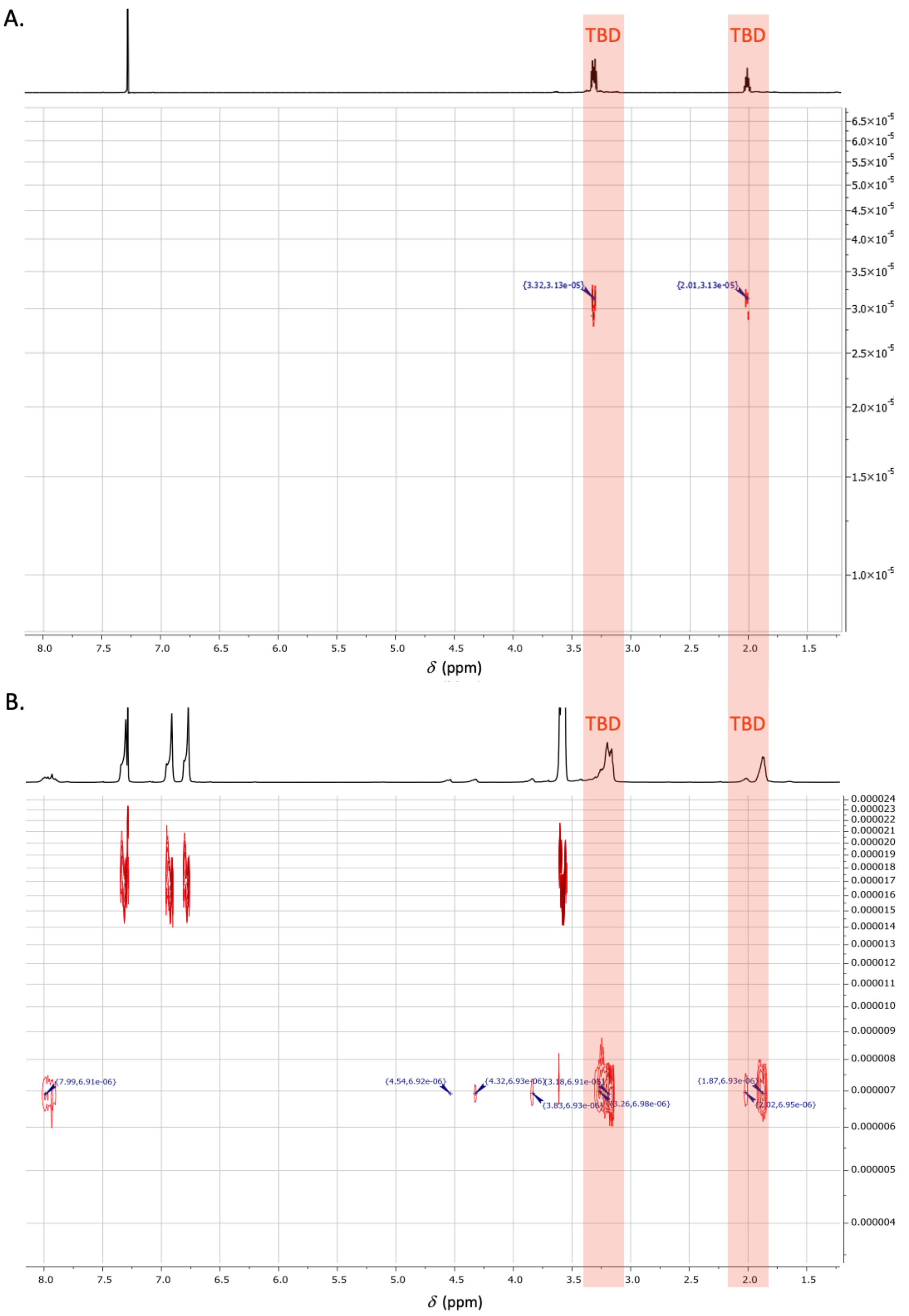


**Figure S7.** DOSY NMR experiments of **A.** 1 eq. PET in 10 eq. of Methylimidazole and **B.** 1 eq. of TBD and 1 eq. of PET in 10 eq. of 1-Methylimidazole. (400 MHz, $CDCl_3$, 298 K)

## Kinetics of the depolymerisation reactions with various catalytic loads

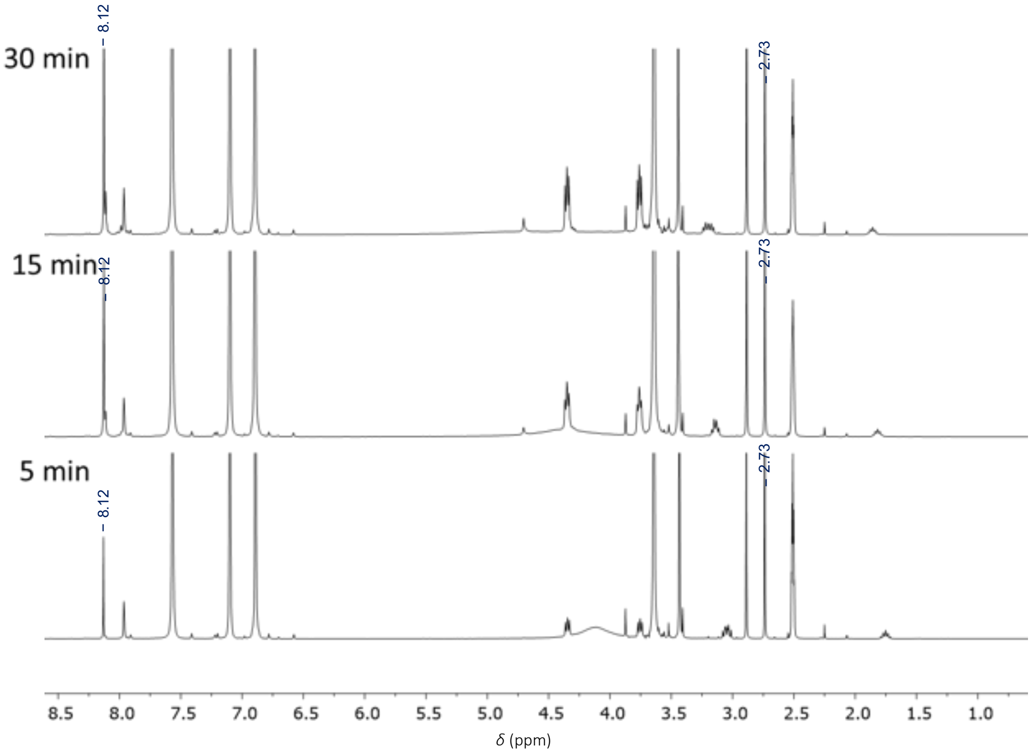


**Figure S8.** Kinetics of PET glycolysis with 3 eq. of ethylene glycol as reagent, **0.1 eq. of TBD** as catalyst and 10 eq. of 1-methylimidazole as solvent, at 100 °C. (400 MHz, DMSO-$d_6$, 298 K) The BHET yield was evaluated by comparing the integration of its characteristic signals ($\delta$ = 8.12 ppm (s, 4H, CH)) with the characteristic signals of DMF as internal standard ($\delta$ = 2.73 ppm (s, 3H, $CH_3$)).

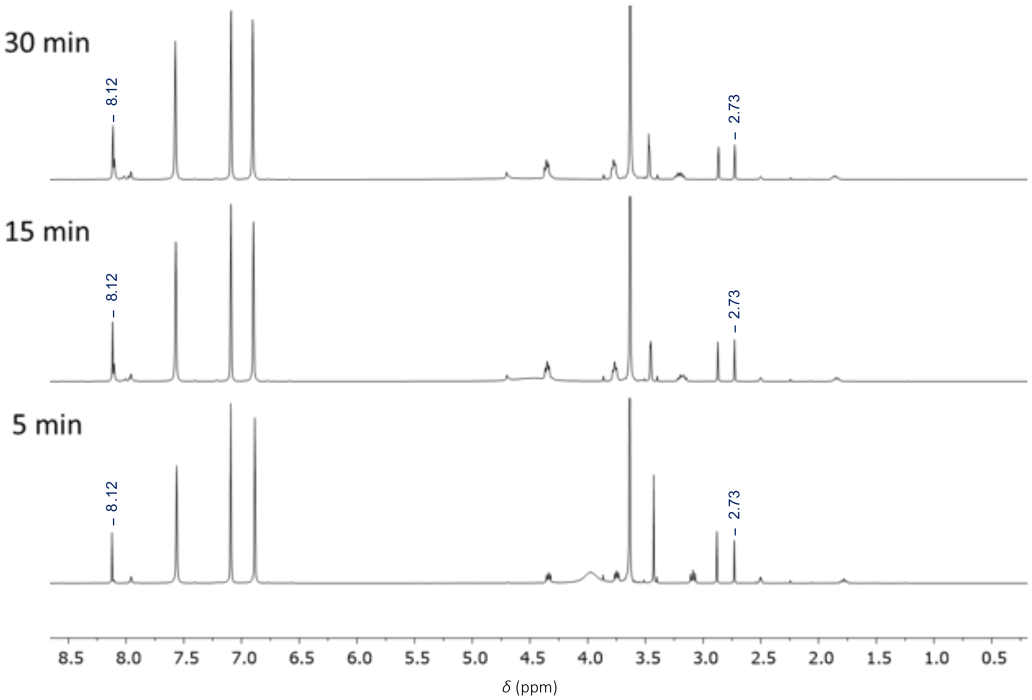


**Figure S9.** Kinetics of PET glycolysis with 3 eq. of ethylene glycol as reagent, **0.2 eq. of TBD** as catalyst and 10 eq. of 1-methylimidazole as solvent, at 100 °C. (400 MHz, DMSO-$d_6$, 298 K) The BHET yield was evaluated by comparing the integration of its characteristic signals ($\delta$ = 8.12 ppm (s, 4H, CH)) with the characteristic signals of DMF as internal standard ($\delta$ = 2.73 ppm (s, 3H, $CH_3$)).

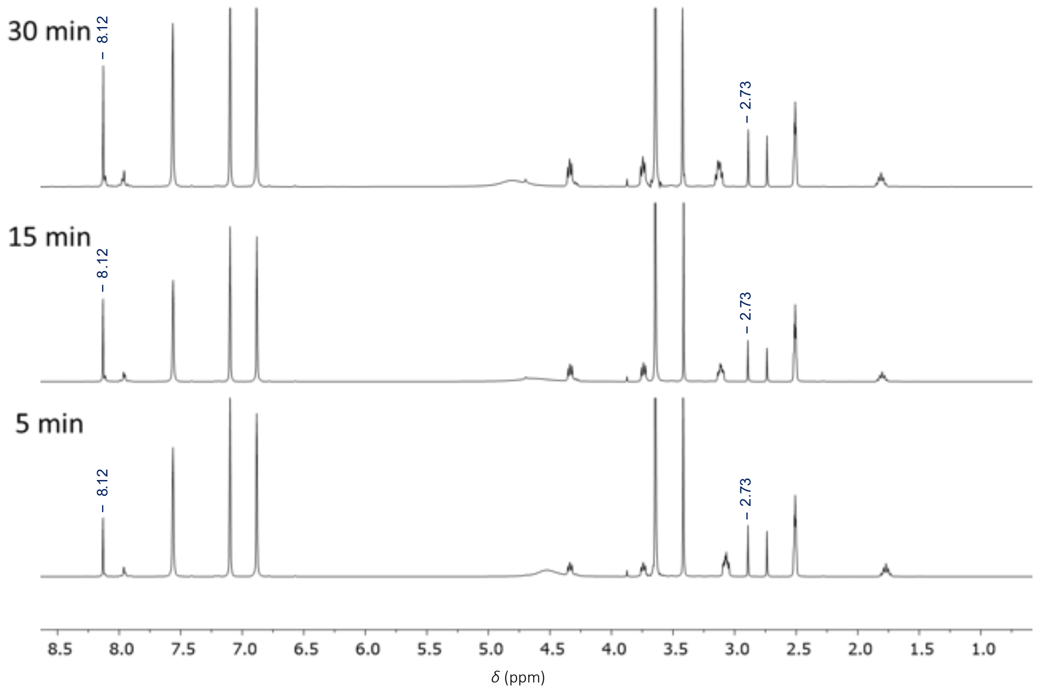


**Figure S10.** Kinetics of PET glycolysis with 3 eq. of ethylene glycol as reagent, **0.5 eq. of TBD** as catalyst and 10 eq. of 1-methylimidazole as solvent, at 100 °C. (400 MHz, DMSO-$d_6$, 298 K) The BHET yield was evaluated by comparing the integration of its characteristic signals ($\delta$ = 8.12 ppm (s, 4H, CH)) with the characteristic signals of DMF as internal standard ($\delta$ = 2.73 ppm (s, 3H, $CH_3$)).

# PET depolymerisation reactions in the presence of water

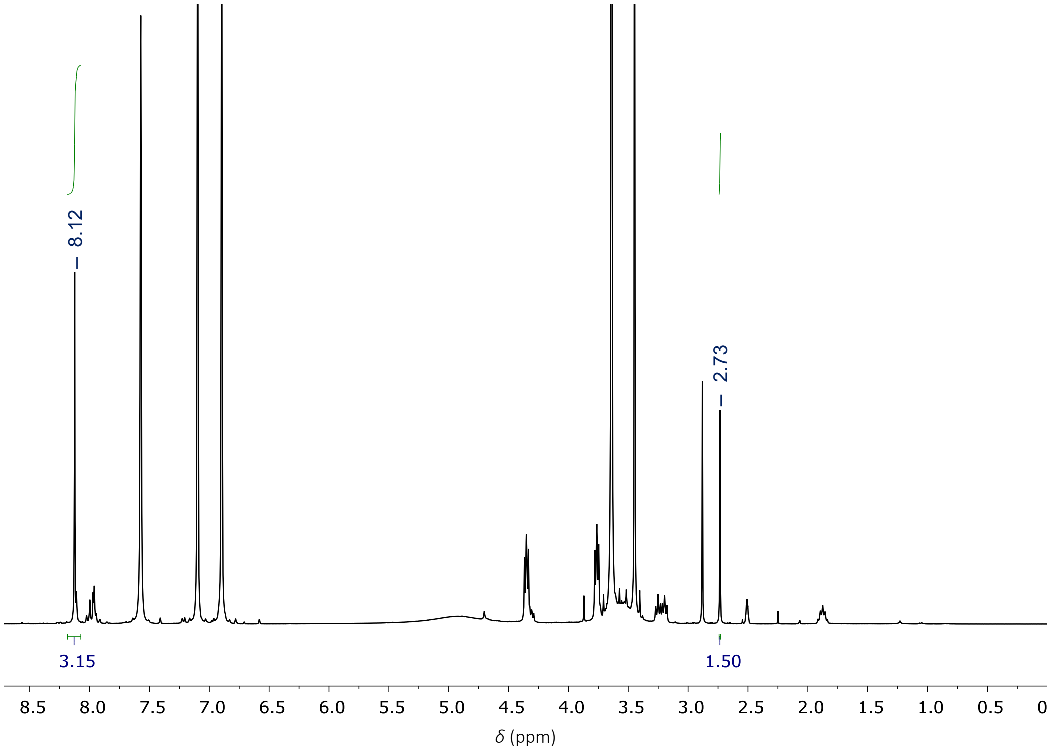


**Figure S11.** $^{1}$H NMR spectrum of PET glycolysis with 3 eq. of ethylene glycol as reagent, 0.2 eq. of TBD as catalyst and 10 eq. of 1-methylimidazole as solvent **in the absence of water**, at 100 °C. (400 MHz, DMSO-$d_6$, 298 K) The BHET yield was evaluated by comparing the integration of its characteristic signals ($\delta$ = 8.12 ppm (s, 4H, CH)) with the characteristic signals of DMF as internal standard ($\delta$ = 2.73 ppm (s, 3H, $CH_3$)).

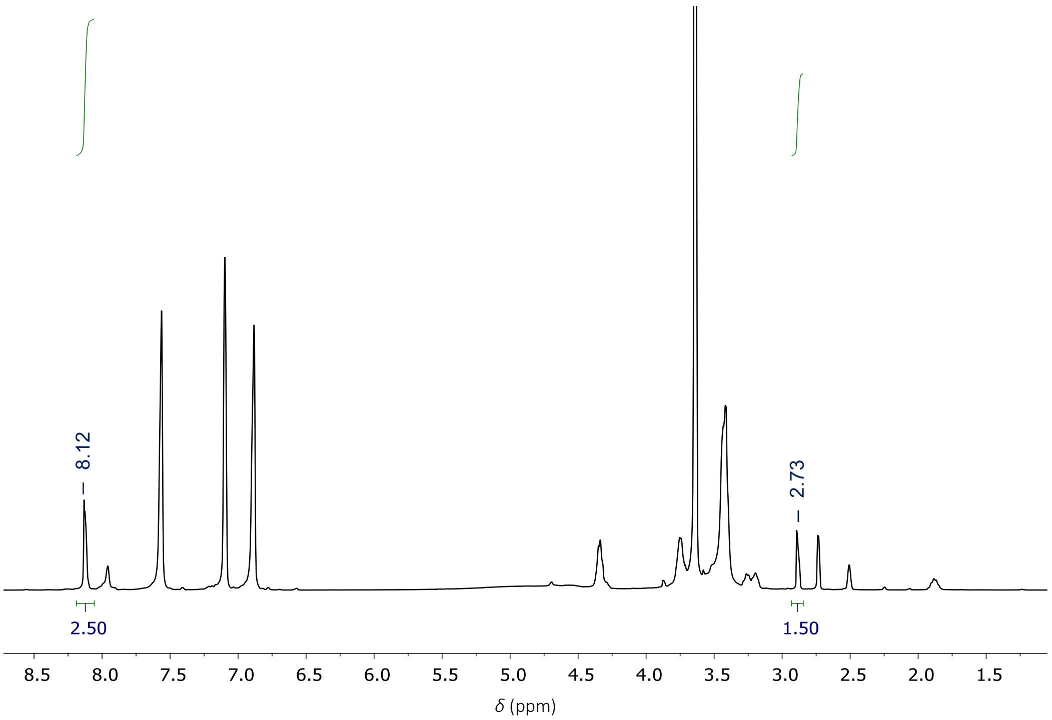


**Figure S12.** $^{1}$H NMR spectrum of PET glycolysis with 3 eq. of ethylene glycol as reagent, 0.2 eq. of TBD as catalyst and 10 eq. of 1-methylimidazole as solvent **in the presence of 0.5 eq. of water**, at 100 °C. (400 MHz, DMSO-$d_6$, 298 K) The BHET yield was evaluated by comparing the integration of its characteristic signals ($\delta$ = 8.12 ppm (s, 4H, CH)) with the characteristic signals of DMF as internal standard ($\delta$ = 2.73 ppm (s, 3H, $CH_3$)).

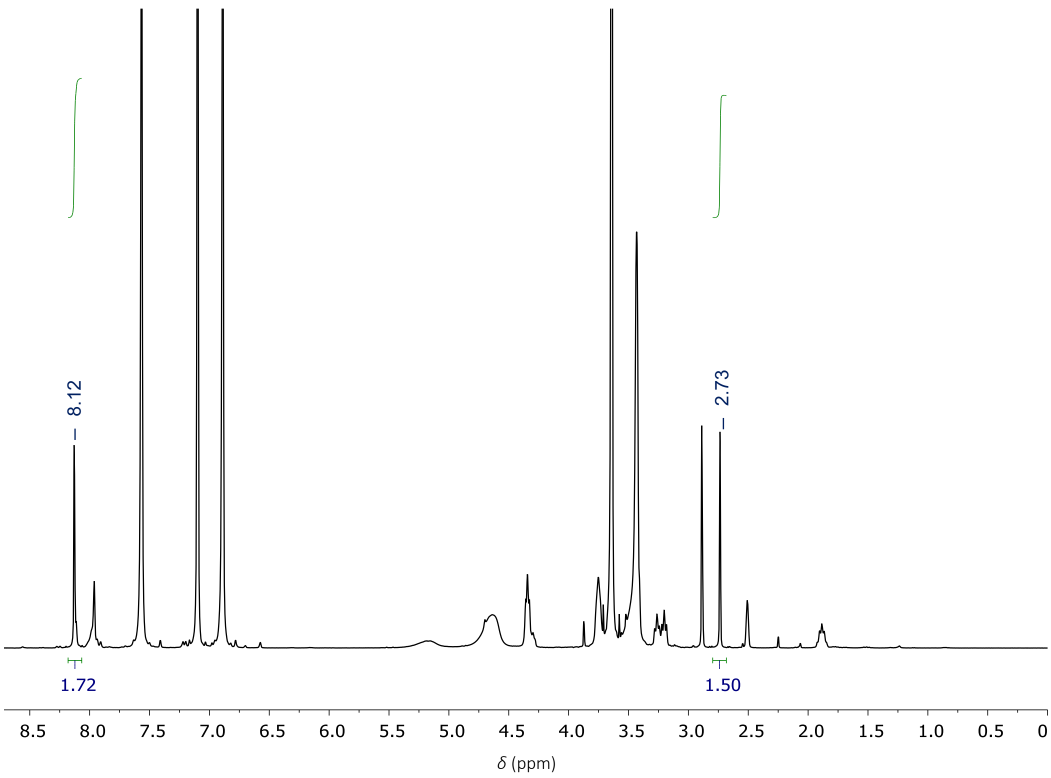


**Figure S13.** $^1$H NMR spectrum of PET glycolysis with 3 eq. of ethylene glycol as reagent, 0.2 eq. of TBD as catalyst and 10 eq. of 1-methylimidazole as solvent **in the presence of 1 eq. of water**, at 100 °C. (400 MHz, DMSO-$d_6$, 298 K) The BHET yield was evaluated by comparing the integration of its characteristic signals ($\delta$ = 8.12 ppm (s, 4H, CH)) with the characteristic signals of DMF as internal standard ($\delta$ = 2.73 ppm (s, 3H, $CH_3$)).

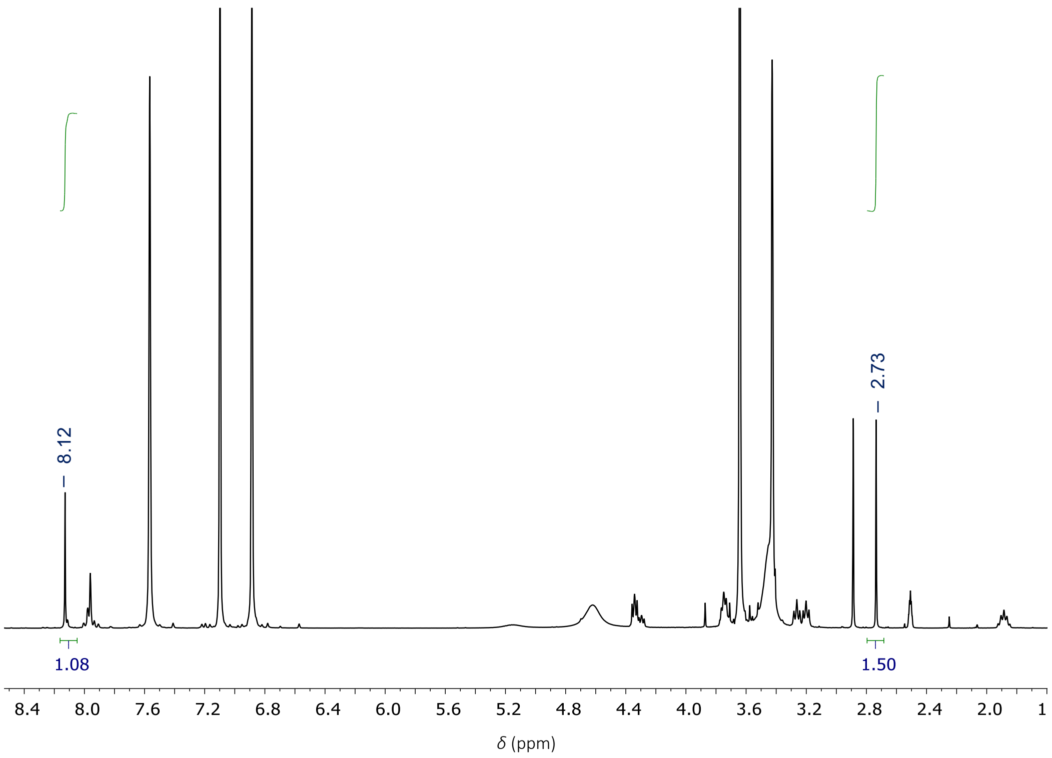


**Figure S14.** $^1$H NMR spectrum of PET glycolysis with 3 eq. of ethylene glycol as reagent, 0.2 eq. of TBD as catalyst and 10 eq. of 1-methylimidazole as solvent **in the presence of 3 eq. of water**, at 100 °C. (400 MHz, DMSO-$d_6$, 298 K) The BHET yield was evaluated by comparing the integration of its characteristic signals ($\delta$ = 8.12 ppm (s, 4H, CH)) with the characteristic signals of DMF as internal standard ($\delta$ = 2.73 ppm (s, 3H, $CH_3$)).

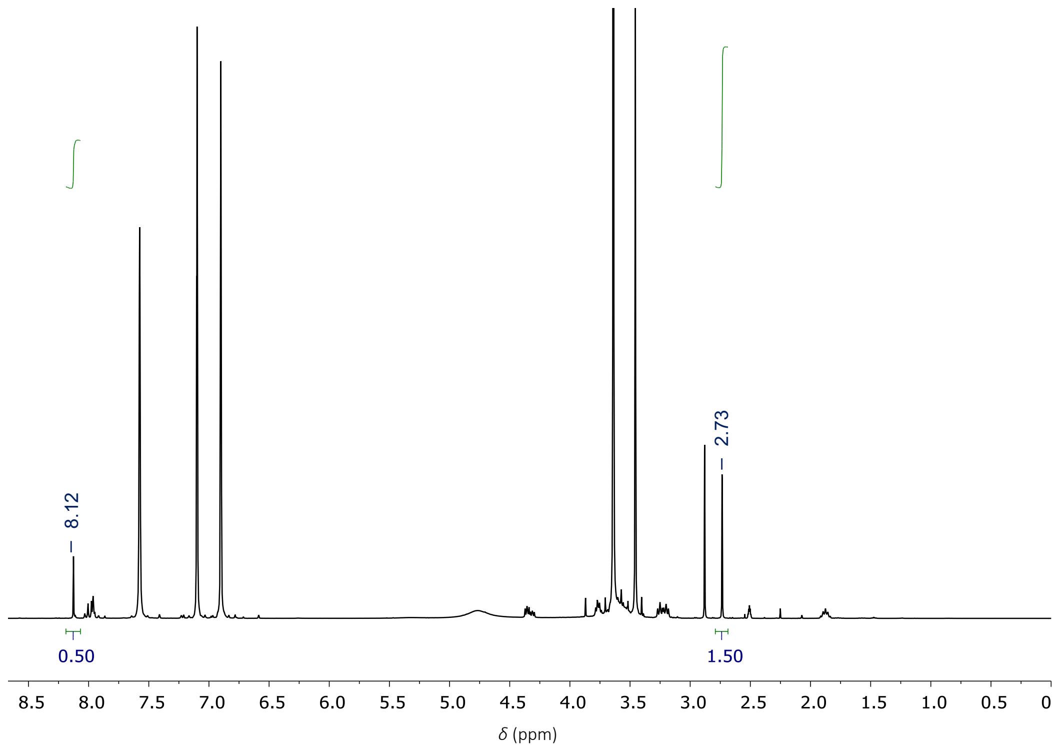


**Figure S15.** $^{1}$H NMR spectrum of PET glycolysis with 3 eq. of ethylene glycol as reagent, 0.2 eq. of TBD as catalyst and 10 eq. of 1-methylimidazole as solvent **in the presence of 5 eq. of water**, at 100 °C. (400 MHz, DMSO-$d_6$, 298 K) The BHET yield was evaluated by comparing the integration of its characteristic signals ($\delta$ = 8.12 ppm (s, 4H, CH)) with the characteristic signals of DMF as internal standard ($\delta$ = 2.73 ppm (s, 3H, $CH_3$)).

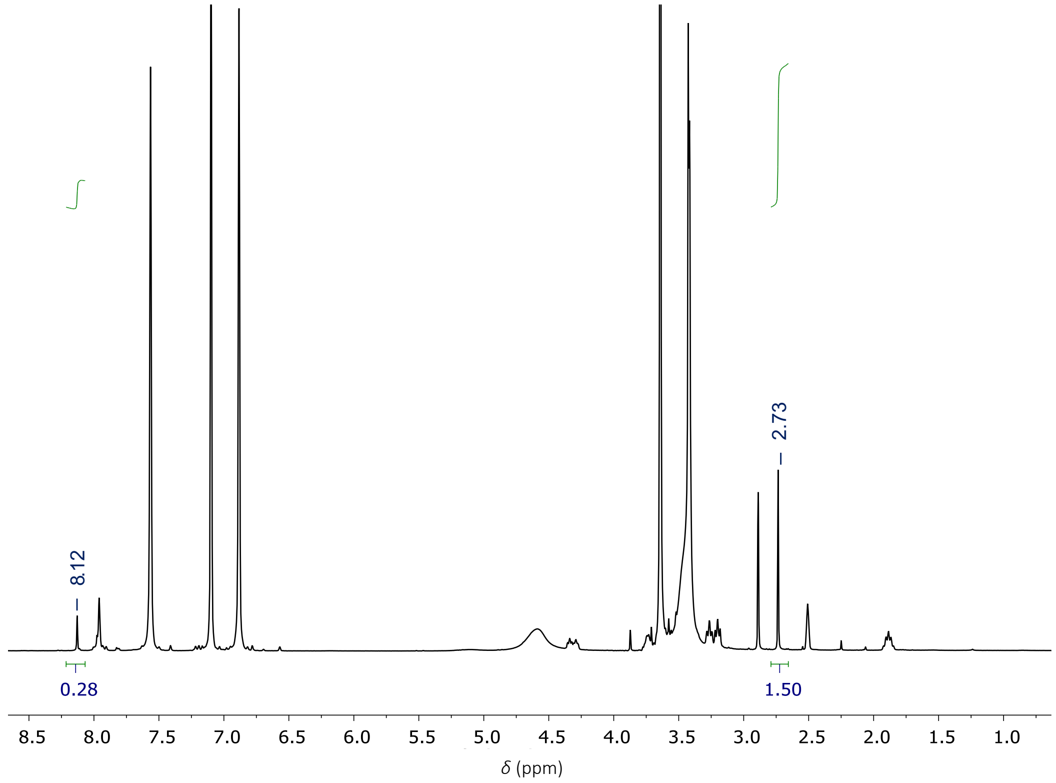


**Figure S16.** $^{1}$H NMR spectrum of PET glycolysis with 3 eq. of ethylene glycol as reagent, 0.2 eq. of TBD as catalyst and 10 eq. of 1-methylimidazole as solvent **in the presence of 10 eq. of water**, at 100 °C. (400 MHz, DMSO-$d_6$, 298 K) The BHET yield was evaluated by comparing the integration of its characteristic signals ($\delta$ = 8.12 ppm (s, 4H, CH)) with the characteristic signals of DMF as internal standard ($\delta$ = 2.73 ppm (s, 3H, $CH_3$)).

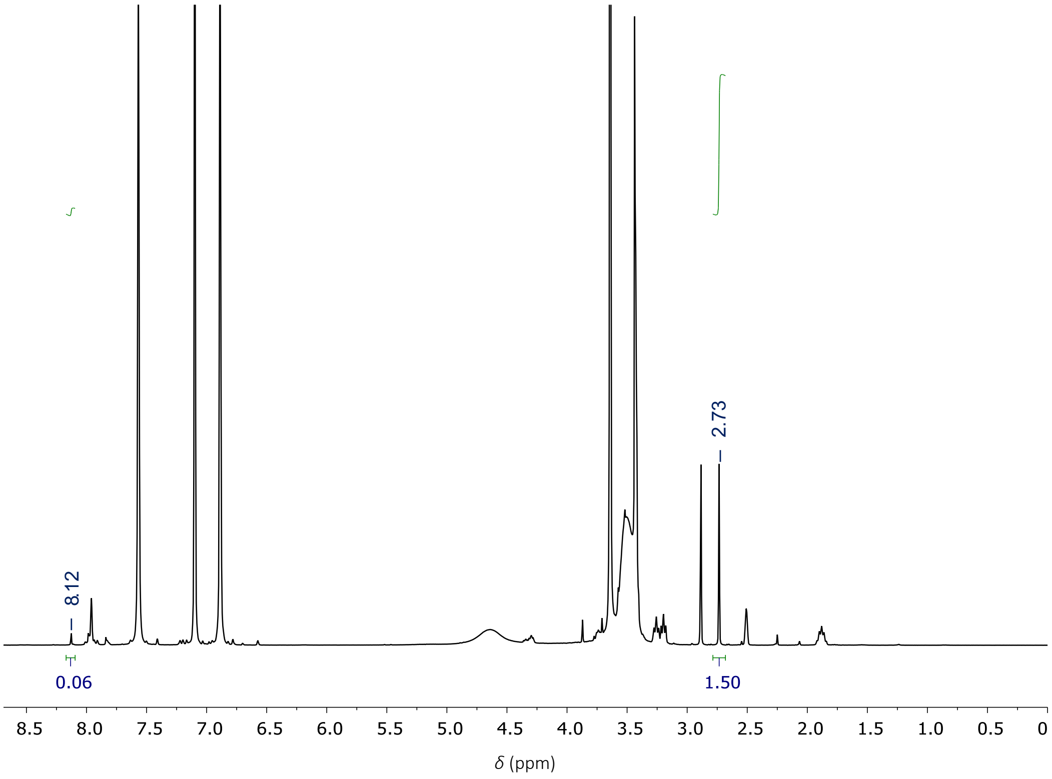


**Figure S17.** $^{1}H$ NMR spectrum of PET glycolysis with 3 eq. of ethylene glycol as reagent, 0.2 eq. of TBD as catalyst and 10 eq. of 1-methylimidazole as solvent **in the presence of 20 eq. of water**, at 100 °C. (400 MHz, DMSO-$d_6$, 298 K) The BHET yield was evaluated by comparing the integration of its characteristic signals ($\delta$ = 8.12 ppm (s, 4H, CH)) with the characteristic signals of DMF as internal standard ($\delta$ = 2.73 ppm (s, 3H, $CH_3$)).

# Kinetics for the depolymerisation reactions involving tBuOK

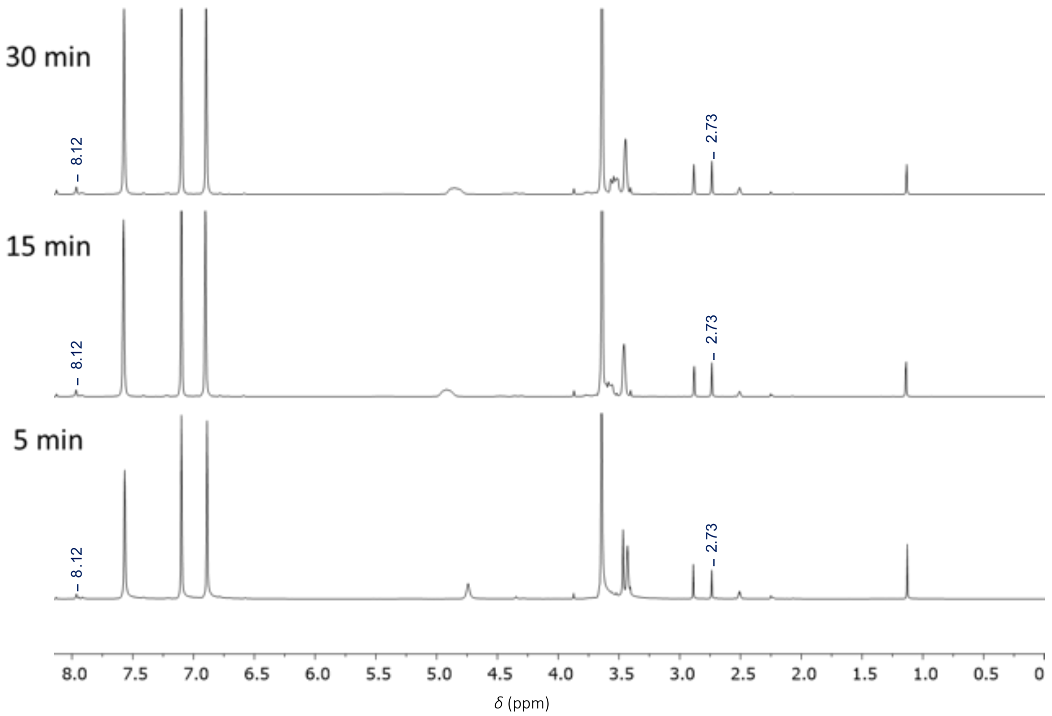


**Figure S18.** Kinetics of PET glycolysis with 3 eq. of ethylene glycol as reagent, **0.2 eq. of tBuOK as catalyst** and 10 eq. of 1-methylimidazole as solvent, at 100 °C. (400 MHz, DMSO-$d_6$, 298 K) The BHET yield was evaluated by comparing the integration of its characteristic signals ($\delta$ = 8.12 ppm (s, 4H, CH)) with the characteristic signals of DMF as internal standard ($\delta$ = 2.73 ppm (s, 3H, $CH_3$)).

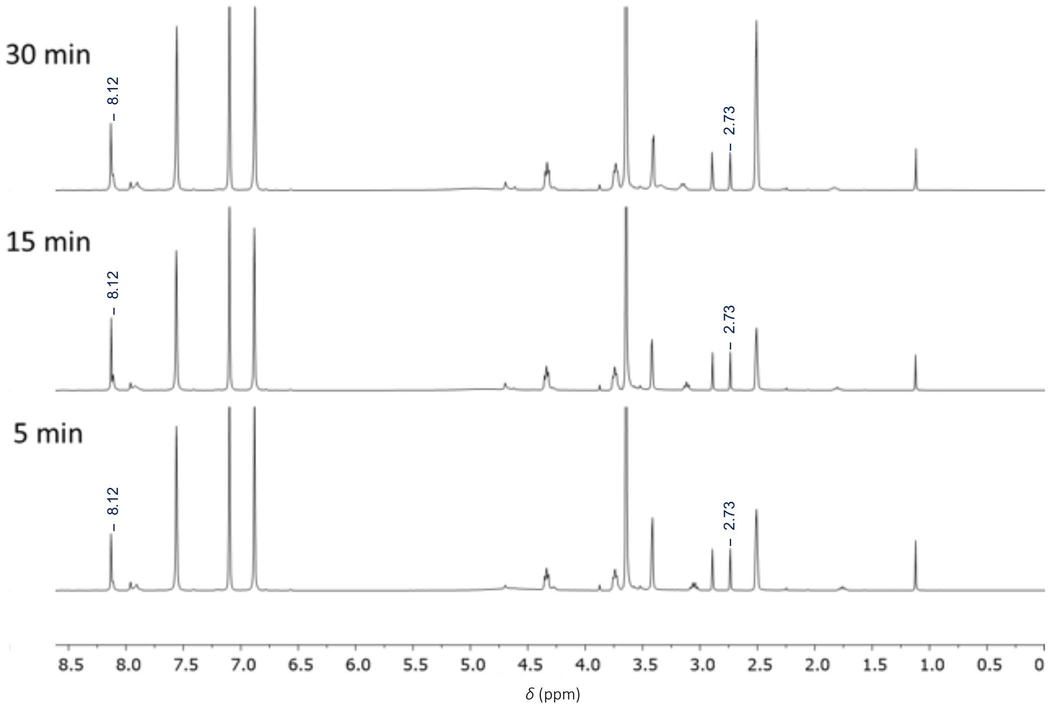


**Figure S19**. Kinetics of PET glycolysis with 3 eq. of ethylene glycol as reagent, **0.1 eq of TBD and 0.2 eq. of tBuOK as catalyst** and 10 eq. of 1-methylimidazole as solvent, at 100 °C. (400 MHz, DMSO-$d_6$, 298 K) The BHET yield was evaluated by comparing the integration of its characteristic signals ($\delta$ = 8.12 ppm (s, 4H, CH)) with the characteristic signals of DMF as internal standard ($\delta$ = 2.73 ppm (s, 3H, $CH_3$)).

## NMR spectra of the crude product for the depolymerisation reactions on complex wastes

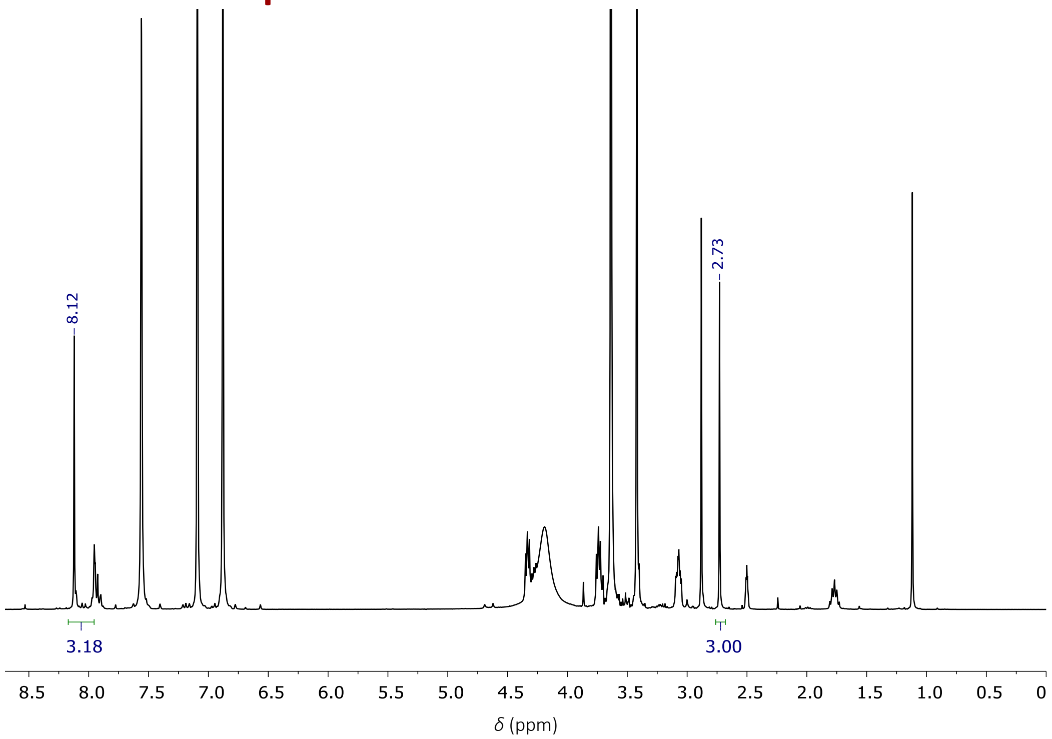


**Figure S20.** $^{1}$H NMR spectrum of the crude product for the depolymerisation of a mixture **of PET and aluminium (1:1 in weight)** with 3 eq. of ethylene glycol as reagent, **0.1 eq. of TBD as catalyst** and 10 eq. of 1-methylimidazole as solvent at 100 °C. (300 MHz, DMSO-$d_6$, 298 K) The BHET yield was evaluated by comparing the integration of its characteristic signals ($\delta$ = 8.12 ppm (s, 4H, CH)) with the characteristic signals of DMF as internal standard ($\delta$ = 2.73 ppm (s, 3H, $CH_3$)).

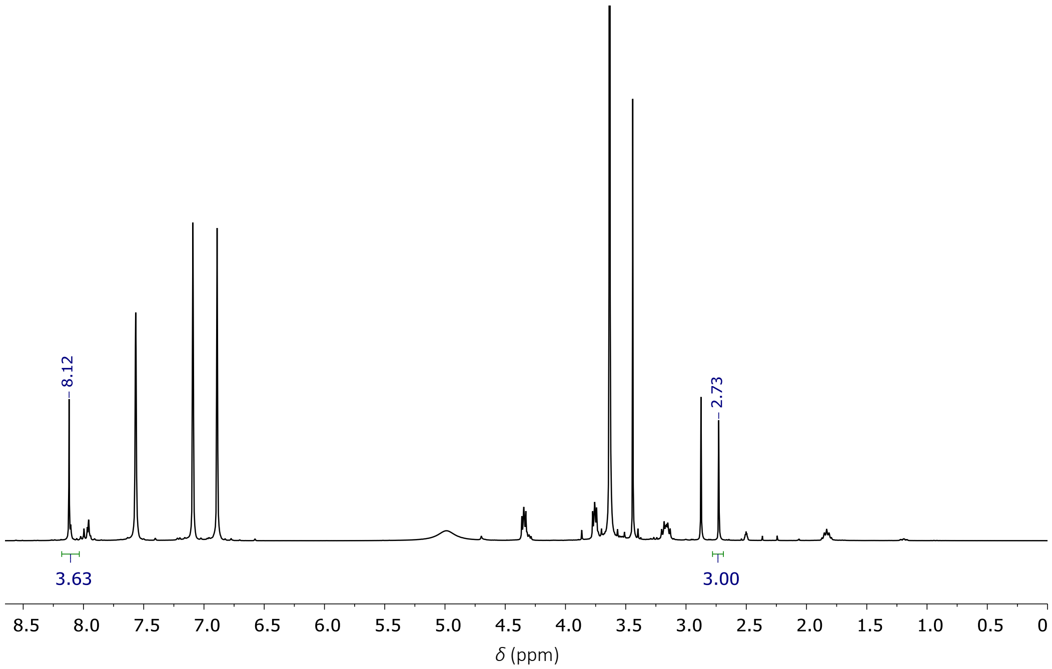


**Figure S21.** $^{1}$H NMR spectrum of the crude product for the depolymerisation of a mixture **of PET and aluminium (1:1 in weight)** with 3 eq. of ethylene glycol as reagent, **0.1 eq. of TBD and 0.2 eq. of tBuOK as catalyst,** and 10 eq. of 1-methylimidazole as solvent at 100 °C. (300 MHz, DMSO-$d_6$, 298 K) The BHET yield was evaluated by comparing the integration of its characteristic signals ($\delta$ = 8.12 ppm (s, 4H, CH)) with the characteristic signals of DMF as internal standard ($\delta$ = 2.73 ppm (s, 3H, $CH_3$)).

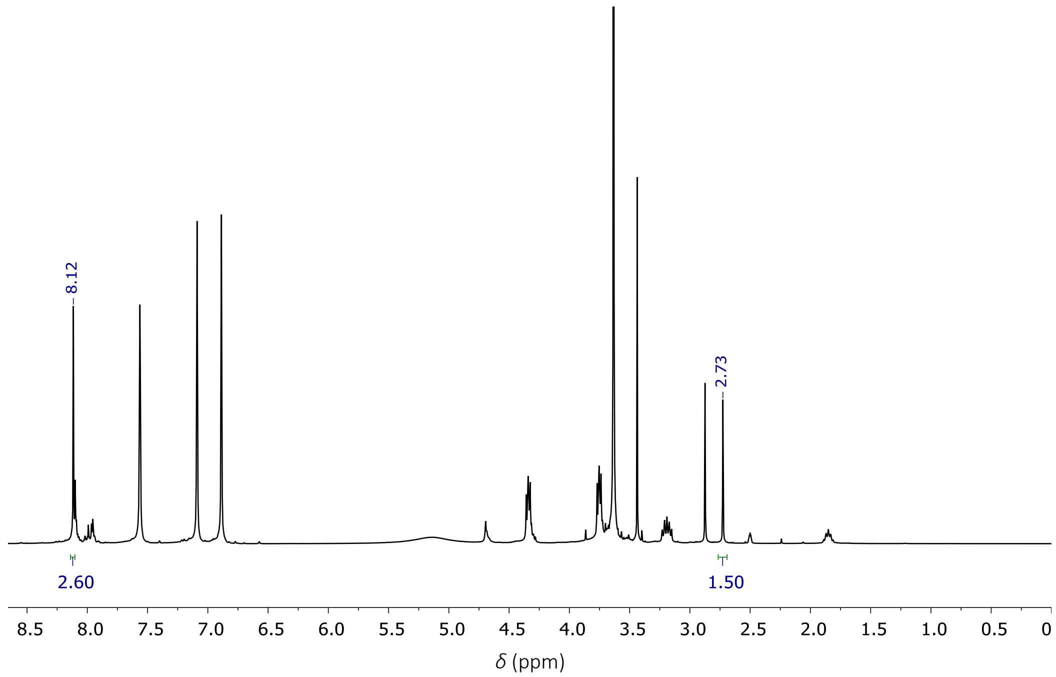


**Figure S22.** $^{1}$H NMR spectrum of the crude product for the depolymerisation of **coloured PET** with 3 eq. of ethylene glycol as reagent, **0.1 eq. of TBD as catalyst** and 10 eq. of 1-methylimidazole as solvent at 100 °C. (300 MHz, DMSO-$d_6$, 298 K) The BHET yield was evaluated by comparing the integration of its characteristic signals ($\delta$ = 8.12 ppm (s, 4H, CH)) with the characteristic signals of DMF as internal standard ($\delta$ = 2.73 ppm (s, 3H, $CH_3$)).

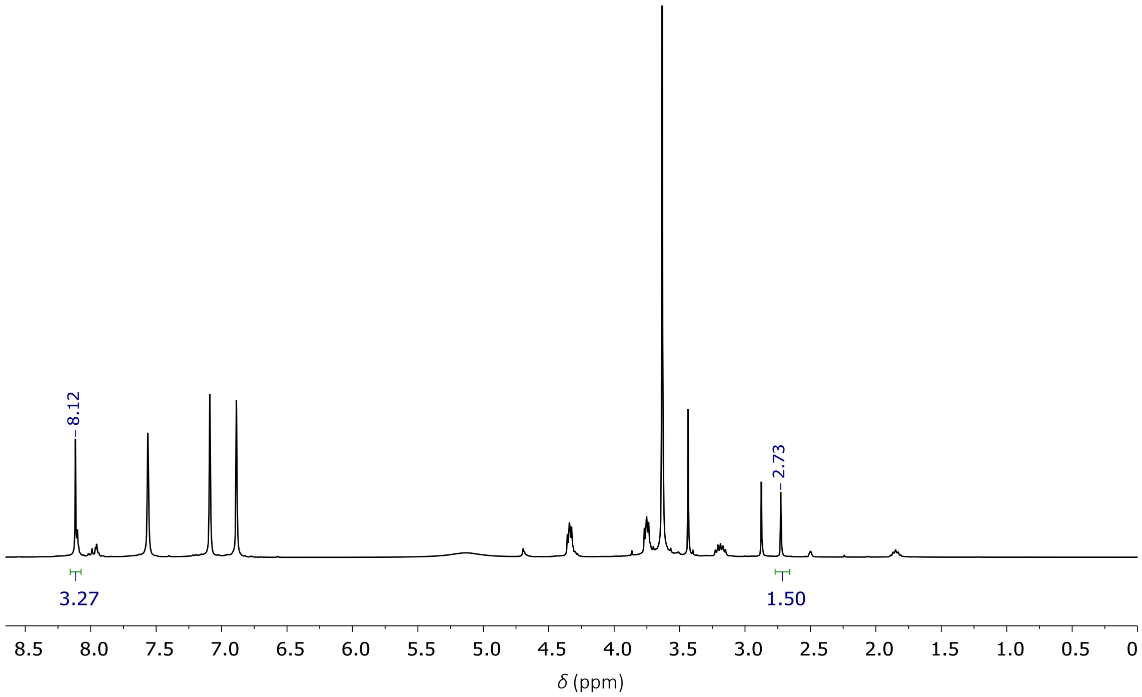


**Figure S23.** $^{1}$H NMR spectrum of the crude product for the depolymerisation of **coloured PET** with 3 eq. of ethylene glycol as reagent, **0.1 eq. of TBD and 0.2 eq. of tBuOK as catalyst** and 10 eq. of 1-methylimidazole as solvent at 100 °C. (300 MHz, DMSO-$d_6$, 298 K) The BHET yield was evaluated by comparing the integration of its characteristic signals ($\delta$ = 8.12 ppm (s, 4H, CH)) with the characteristic signals of DMF as internal standard ($\delta$ = 2.73 ppm (s, 3H, $CH_3$)).

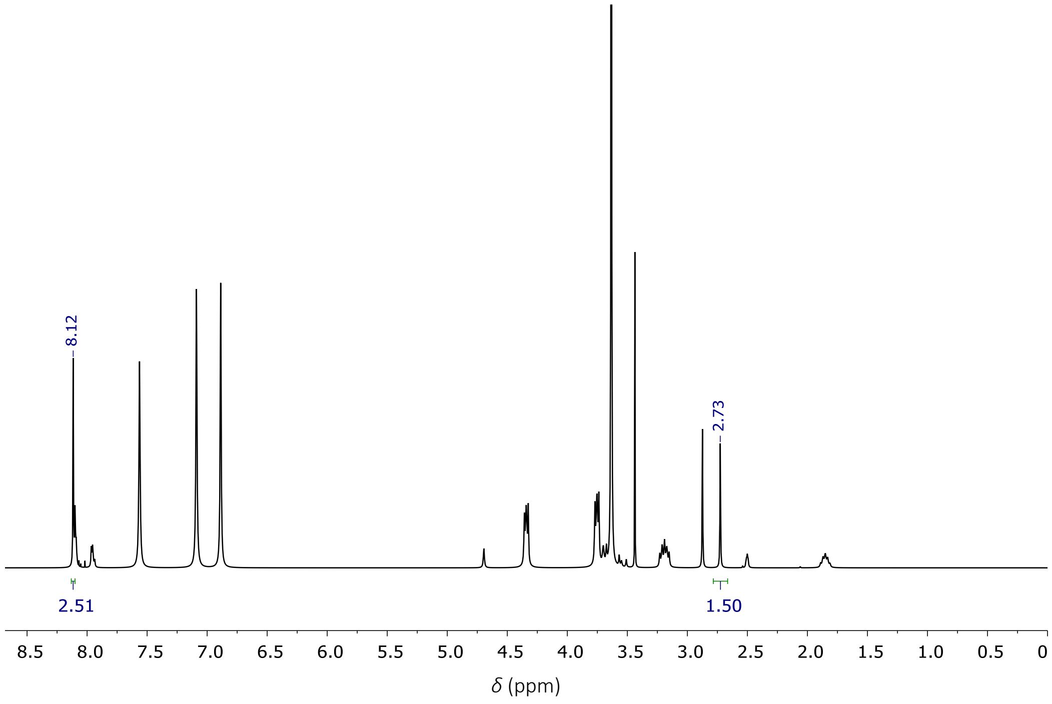


**Figure S24**. $^{1}$H NMR spectrum of the crude product for the depolymerisation of **PET covered with a layer of PE** with 3 eq. of ethylene glycol as reagent, **0.1 eq. of TBD as catalyst** and 10 eq. of 1-methylimidazole as solvent at 100 °C. (300 MHz, DMSO-$d_6$, 298 K) The BHET yield was evaluated by comparing the integration of its characteristic signals ($\delta$ = 8.12 ppm (s, 4H, CH)) with the characteristic signals of DMF as internal standard ($\delta$ = 2.73 ppm (s, 3H, $CH_3$)).

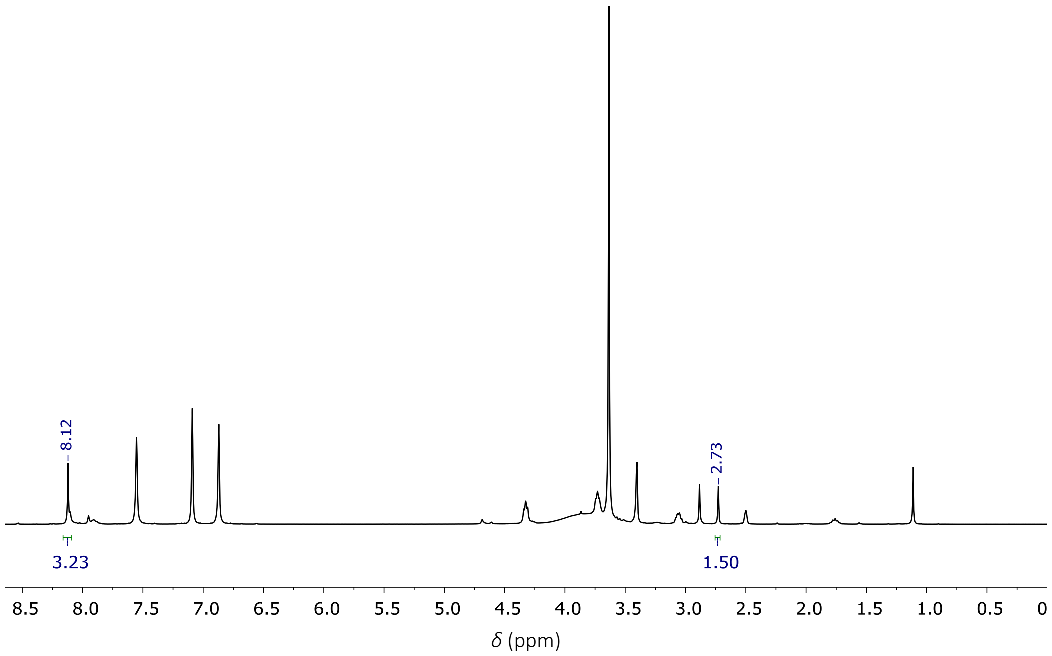


**Figure S25.** $^{1}$H NMR spectrum of the crude product for the depolymerisation of **PET covered with a layer of PE** with 3 eq. of ethylene glycol as reagent, **0.1 eq. of TBD and 0.2 eq. of tBuOK as catalyst** and 10 eq. of 1-methylimidazole as solvent at 100 °C. (300 MHz, DMSO-$d_6$, 298 K) The BHET yield was evaluated by comparing the integration of its characteristic signals ($\delta$ = 8.12 ppm (s, 4H, CH)) with the characteristic signals of DMF as internal standard ($\delta$ = 2.73 ppm (s, 3H, $CH_3$)).

# $^{1}$H NMR spectra of isolated molecules

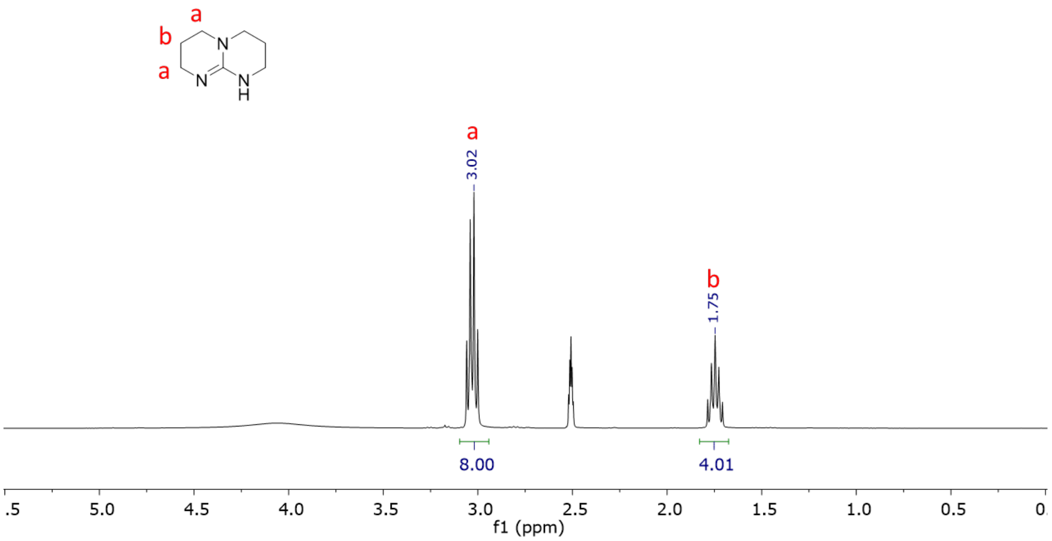


Figure S26. $^{1}$H NMR spectrum of pure TBD in its free base form (400 MHz, DMSO-$d_6$, 298 K).

**$^{1}$H NMR** (400 MHz, DMSO-d$_6$, 298 K) $\delta$ 3.03 (q, J = 5.8 Hz, 8H), 1.83 – 1.67 (m, 4H).

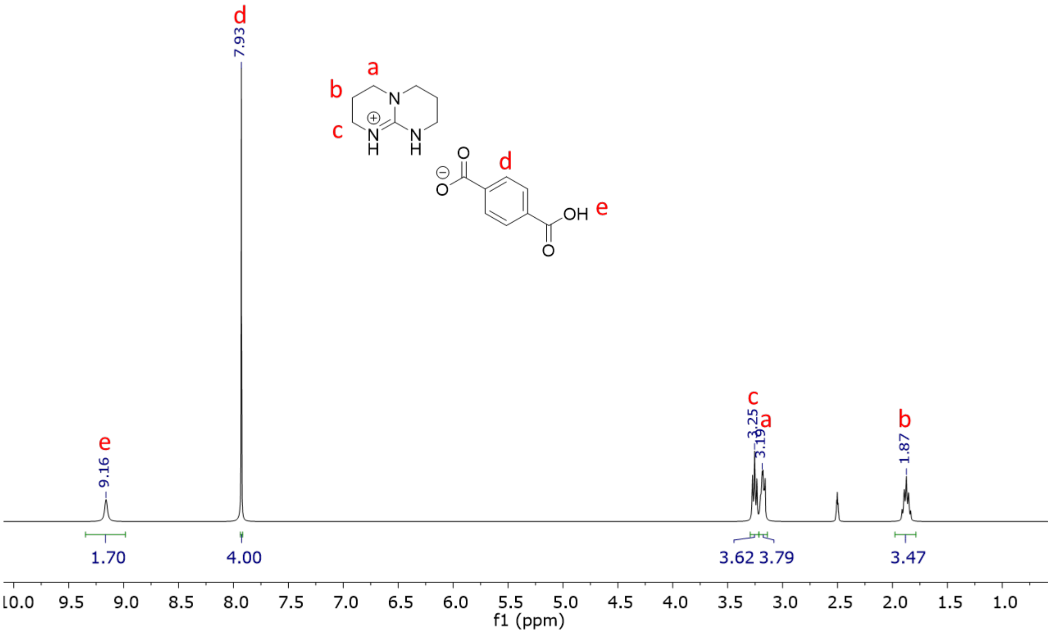


**Figure S27.** $^{1}$H NMR spectrum of TBD:TPA (1:1) (400 MHz, DMSO-$d_6$, 298 K).

**$^{1}$H NMR** (400 MHz DMSO-d$_6$, 298 K) $\delta$ 9.16 (s, 2H), 7.93 (s, 4H), 3.25 (t, J = 5.9 Hz, 4H), 3.21 – 3.14 (t, J = 5.9 Hz, 4H), 1.87 (q, J = 5.9 Hz, 4H).

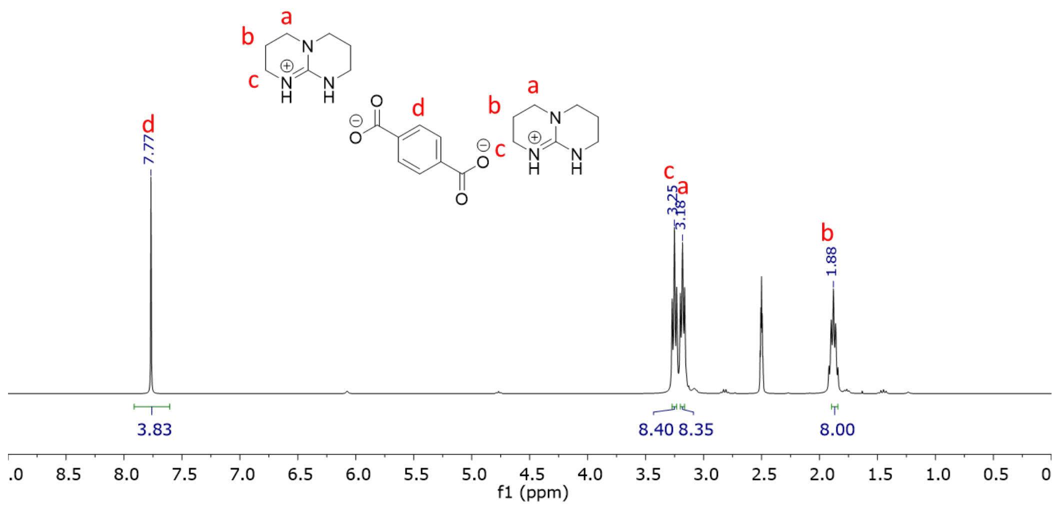


**Figure S28.** $^1$H NMR spectrum of TBD:TPA (1:2) (400 MHz, DMSO-$d_6$, 298 K).

**$^1$H NMR** (400 MHz, DMSO-$d_6$, 298 K) $\delta$ 7.77 (s, 4H), 3.25 (t, J = 5.9 Hz, 4H), 3.21 – 3.16 (t, 4H), 1.88 (q, J = 5.9 Hz, 4H).

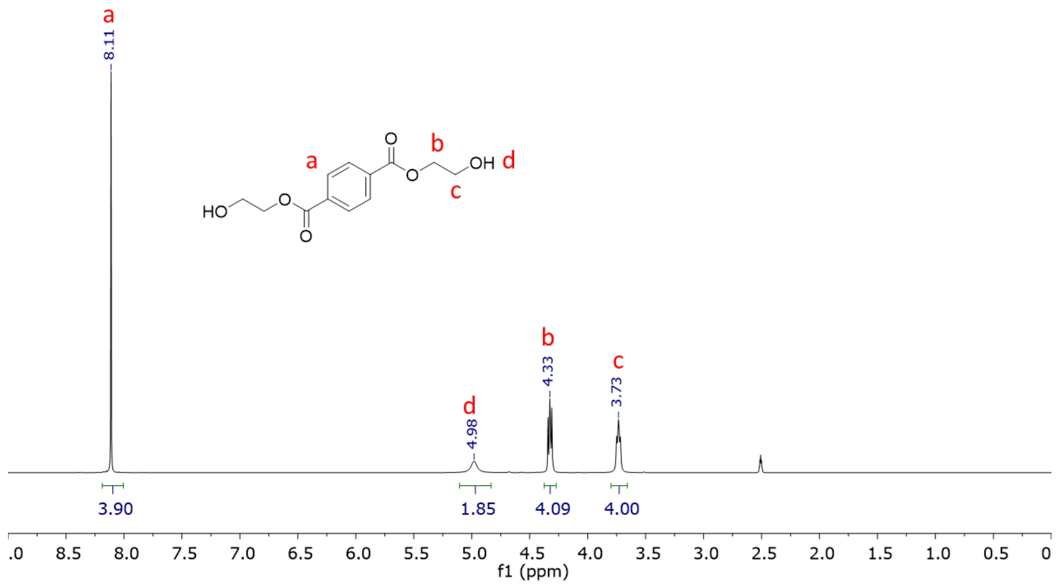


Figure S29. $^1$H NMR spectrum of obtained BHET product (400 MHz, DMSO-$d_6$, 298 K).

**$^1$H NMR** (400 MHz, DMSO-$d_6$, 298 K) $\delta$ 8.11(s, 4H), 4.98(s, 2H), 4.33(t, 4H), 3.73(t, 4H).
